# Genome-scale CRISPR screen reveals neddylation to contribute to cisplatin resistance of testicular germ cell tumours

**DOI:** 10.1038/s41416-023-02247-5

**Published:** 2023-04-06

**Authors:** Kai Funke, Ulf Einsfelder, Aylin Hansen, Lena Arévalo, Simon Schneider, Daniel Nettersheim, Valentin Stein, Hubert Schorle

**Affiliations:** 1 Department of Developmental Pathology, Institute of Pathology, University Hospital Bonn, Bonn, Germany; 2 Institute of Physiology II, University Hospital Bonn, Bonn, Germany; 3 Department of Urology, Urological Research Laboratory, Translational UroOncology, Medical Faculty and University Hospital Düsseldorf, Heinrich Heine University Düsseldorf, Düsseldorf, Germany

**Keywords:** Germ cell tumours, Germ cell tumours

## Abstract

**Background:**

Type II testicular germ cell tumours (TGCT) are the most prevalent tumours in young men. Patients suffering from cisplatin-resistant TGCTs are facing very poor prognosis demanding novel therapeutic options. Neddylation is a known posttranslational modification mediating many important biological processes, including tumorigenesis. Overactivation of the neddylation pathway promotes carcinogenesis and tumour progression in various entities by inducing proteasomal degradation of tumour suppressors (e.g., p21, p27).

**Methods:**

We used a genome-scale CRISPR/Cas9 activation screen to identify cisplatin resistance factors. TGCT cell lines were treated with the neddylation inhibitor (MLN4924)/cisplatin/combination and investigated for changes in viability (XTT assay), apoptosis/cell cycle (flow cytometry) as well as in the transcriptome (3’mRNA sequencing).

**Results:**

*NAE1* overexpression was detected in cisplatin-resistant colonies from the CRISPR screen. Inhibition of neddylation using MLN4924 increased cisplatin cytotoxicity in TGCT cell lines and sensitised cisplatin-resistant cells towards cisplatin. Apoptosis, G2/M-phase cell cycle arrest, γH2A.X/P27 accumulation and mesoderm/endoderm differentiation were observed in TGCT cells, while fibroblast cells were unaffected.

**Conclusions:**

We identified overactivation of neddylation as a factor for cisplatin resistance in TGCTs and highlighted the additive effect of NAE1 inhibition by MLN4924 in combination with cisplatin as a novel treatment option for TGCTs.

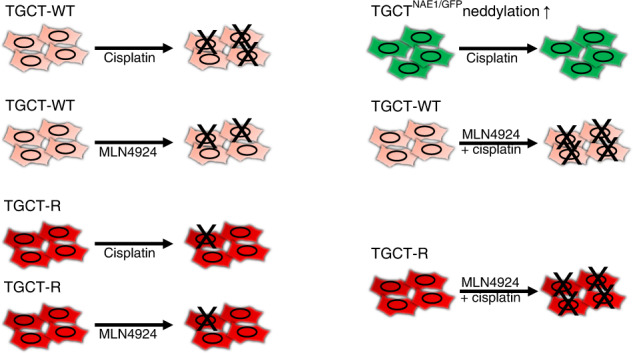

## Background

In Western countries over the past decades, incidence of testicular germ cell tumours (TGCT) is rising. Type II TGCTs represent the tumour entity with the highest prevalence in young men aged 20–40 years [1, 2] arising from primordial germ cells (PGCs), which arrest in early development and forming a germ cell neoplasia in situ (GCNIS). GCNIS eventually progress to seminomas and non-seminomas [3–5]. Due to high similarity in global gene expression, epigenetic profile and histology with PGCs, seminomas are viewed as default pathway of TGCT development [6]. Non-seminomas are classified into embryonal carcinomas (EC), choriocarcinomas (CC), yolk-sac tumours (YST) and teratomas (Ter). ECs express markers of totipotency and are therefore considered as type II TGCT stem cell population, which is able to differentiate into CC, YST and Ter [4, 7, 8]. It has been shown that seminomas are able to reprogramme in a somatic microenvironment towards an EC-like cell fate [9, 10]. While 95% of patients with TGCTs are cured by orchiectomy and subsequent cisplatin-based chemotherapy, ~20% of metastatic non-seminoma patients develop cisplatin resistance facing poor prognosis due to lack of further treatment options [2, 11, 12]. Several cisplatin resistance factors have already been described [13–15] and grouped in pre-, on-, post- and off-target effects [15]. Among others, altered cellular drug uptake/efflux by deregulation of copper transporter 1 and *ATP7A*/*ATP7B* expression (pre-target) [16], increased levels of cytoplasmatic scavenger proteins like glutathione (pre-target) [17], upregulated DNA repair systems instead of apoptosis initiation (on-target) [18], overactivated PI3K/AKT signalling supporting p21 induced cell cycle arrest (off-target) as well as upregulated *MDM2* expression resulting in p53 degradation thereby avoiding cell death (post-target) [19, 20] were described. All these factors emphasize that cisplatin resistance is multifactorial and cannot be reduced to one key mechanism [15] requiring more research to identify further cisplatin resistance factors in order to reveal potential alternative treatment options for TGCTs.

**Fig. 1:**
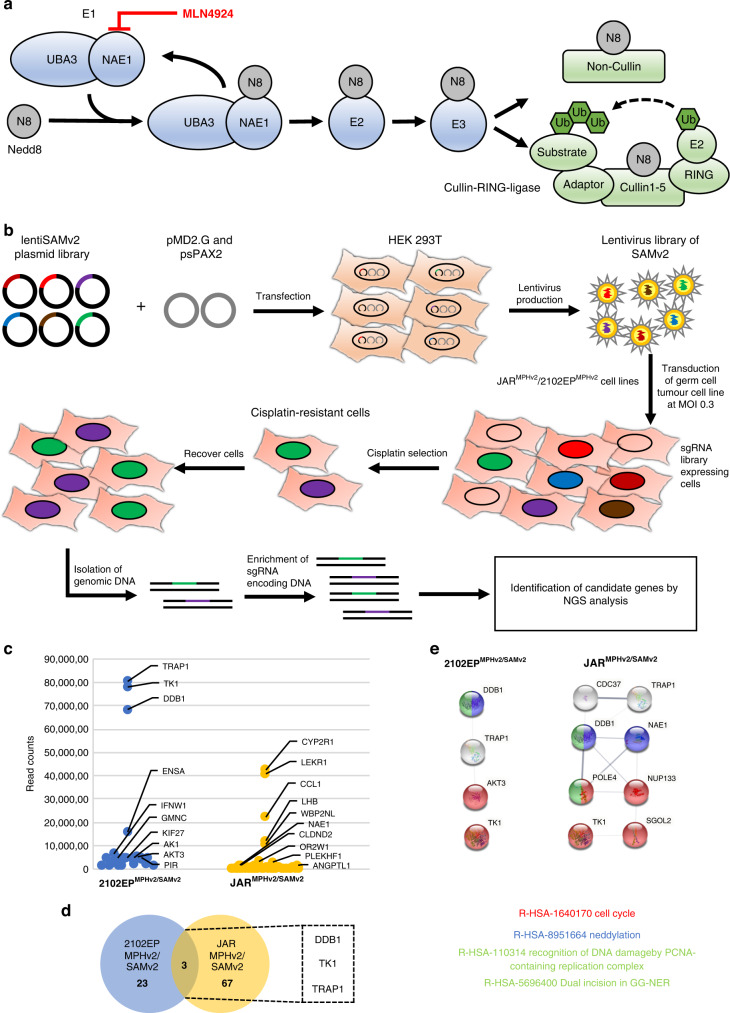
CRISPR/Cas9 screen for cisplatin resistance factors. **a** Illustration of the neddylation pathway. NEDD8 is conjugated to an E1 complex and transferred in a canonical manner via E2 and E3 ligases to the substrates which are divided into cullins and non-cullins. MLN4924 inhibits neddylation cascade via NAE1 binding. **b** Schematic representation of the CRISPR/Cas9 screen using 2102EP and JAR cells. Lentiviral particles were produced in HEK293T cells based on the lentiSAMv2 (activation approach) plasmid library. For the genome-scale activation screen, a stable JAR^MPHv2^ and 2102EP^MPHv2^ cell line expressing MS2-p65-HSF1 were transduced with the lentiSAMv2 library encoding dCas9-VP64 and sgRNAs. Gene-edited cells were treated with cisplatin, and surviving cells were recovered in cell culture medium. To identify candidate genes genomic DNA was isolated, sgRNA encoding region was amplified and analysed via next-generation sequencing. **c** Identification of candidate genes for cisplatin resistance with a read count >10,000 are depicted for JAR^MPHv2/SAMv2^ and 2102EP^MPHv2/SAMv2^ cells. **d** Venn diagram showing unique and common candidate genes for JAR^MPHv2/SAMv2^ and 2102EP^MPHv2/SAMv2^ cell lines in the activation screen. **e** STRING interaction and Reactome pathway analysis on JAR^MPHv2/SAMv2^ as well as 2102EP^MPHv2/SAMv2^ candidate genes. The CRISPR/Cas9 screen was performed *n* = 1.

The process of neddylation (Fig. 1a) describes the conjugation of the ubiquitin-like small molecule NEDD8 (neuronal precursor cell expressed developmentally downregulated protein 8) to a target protein. Neddylation changes the function, stability or subcellular location of the target protein [21, 22]. NEDD8 is conjugated to its target protein in a canonical manner by initial binding to the E1-NEDD8 activating enzyme (NAE1/UBA3 complex), further transferred via NEDD8-conjugating enzyme E2 (UBE2M or UBE2F) and substrate-specific NEDD8-E3 ligase (RBX1/2, DCN1, etc.) to the final substrates (cullins and non-cullins). Cullins are subunits of the Cullin-RING-ligases (CRL) which are ubiquitin E3 ligases. After neddylation, the CLRs mediate polyubiquitylation of the substrates thereby labelling them for proteasomal degradation. In the cell 20% of all proteasomal degraded proteins are processed via a CLR-based mechanism [23, 24]. Various substrates have been identified including several tumour suppressor proteins like p21, p27, Wee1 and p53 [25]. Overactivation of neddylation leads to increased degradation of these proteins promoting malignant transformation and tumour progression. This has been demonstrated in lung [25], breast [26] and pancreatic cancer [27]. Reduction of neddylation applying the covalent NAE1 inhibitor MLN4924 impaired cell cycle progression and induced apoptosis due to accumulation of p21, p27, Wee1, p53, etc. [25–27]. Further, Zheng et al. were able to show increased cisplatin sensitivity in pancreatic cancer triggered by NAE1 inhibition [27]. To date, 40 clinical trials investigating the potential of MLN4924 in terms of cancer treatment have been enrolled (https://www.clinicaltrials.gov/), indicating versatility and potency of the drug [24].

In this study, we applied a genome-scale CRISPR/Cas9 activation screen on parental TGCT cell lines to generate cisplatin-resistant clones. Investigation of candidate genes allowed us to find and describe pathways leading to cisplatin resistance. We identified upregulation of *NAE1*, a core component of neddylation, to induce cisplatin resistance in 2102EP and JAR cell lines. Application of MLN4924, an inhibitor of neddylation, induced cell cycle arrest and apoptosis in TGCT cell lines. Treatment with both MLN4924 and cisplatin resulted in an additive effect enhancing cell death. Cisplatin-resistant TGCT cell lines were found to revert to cisplatin sensitivity after treatment with MLN4924.

## Materials and methods

### Cell culture

The following cell lines were used: 2102EP, 2102EP-R, NCCIT, NCCIT-R, NT2/D1, NT2/D1-R (all ECs), JAR (choriocarcinoma), TCam-2 (seminoma) and HEK293T (human embryonic kidney). As a control MPAF cells (human adult fibroblast) were included. Cell lines with the suffix “-R” represent cisplatin-resistant sub-lines from the corresponding parental cell line [28, 29]. Cells were cultured as described previously [6, 30, 31]. Short tandem repeat (STR) profiles of all cell lines are checked on a regular basis at the Institute of Forensic Medicine (University Hospital Düsseldorf) and are available upon request (to DN). All cell lines were negative for mycoplasma infection as checked by PCR. Cell line sources are described in Supplementary Methods.

### Genome-scale CRISPR/Cas9 transcriptional activation screen

The screen was performed according to Joung et al. [32]. The human CRISPR activation (SAMv2) plasmid library (Supplementary Table S1) was amplified in Endura ElectroCompetent cells (Lucigen, Middleton, WI, USA). To check the sgRNA coverage, next-generation sequencing using NextSeq™550 (Illumina, San Diego, CA, USA) and Python-/Biopython-based analysis were performed [33–35]. Lentivirus production was performed in HEK293T cells. For concentration Lenti-X-concentrator was used according to the manufacturer’s protocol (Takara BIO INC., Kusatsu, Japan). Multiplicity of Infection (MOI) was determined using transduction via centrifugation and the CellTiter-Glo™ Luminescent Cell Viability Assay Kit (Promega, Madison, WI, USA). This process is described in detail in Supplementary Methods. For the SAM activation approach, 2102EP^MPHv2^ and JAR^MPHv2^ helper cell lines stably expressing p65-HSF1 transactivation domains were generated by transduction with MPHv2 lentivirus. The library transduction (1500 × *g*, 30 min, 32 °C) was performed in six-well cell culture plates (10^6^ cells/well) at MOI of 0.3 (Supplementary Table S2). After 2 days of incubation, including two PBS wash steps (24 and 48 h), cells were transferred to 15 cm cell culture dishes. One day later, cisplatin treatment was applied until all cells in the wild-type control had died.

After recovery of the surviving cells, 2102EP^MPHv2/SAMv2^, JAR^MPHv2/SAMv2^ and corresponding wild-type cells were re-seeded in six-well cell culture plates (0.25 × 10^4^ cells/well). To validate cisplatin resistance, 2102EP, as well as JAR cell lines, were treated with 2.5 µM and 5 µM cisplatin, respectively. Media containing cisplatin was changed every 2nd/3r^d^ day for 2 weeks. The effect on the cells was monitored by bright-field microscopy.

To identify candidate genes, genomic DNA was isolated from 2102EP^MPHv2/SAMv2^ and JAR^MPHv2/SAMv2^ cells (Supplementary Table S2), PCR amplification of the region encoding the sgRNAs was performed with NEBNext^®^ High-Fidelity 2X PCR Master Mix (Ipswich, MA, USA) and obtained PCR products were analysed by next-generation sequencing on the NextSeq™550 device (Illumina). Read counts were analysed using the Python/Biopython-based [33–35] *count_spacers.py* algorithm described by Joung et al. [32]. Further, candidate genes of 2102EP^MPHv2/SAMv2^ and JAR^MPHv2/SAMv2^ with read counts >10,000 were evaluated using the STRING 11.5 database [36]. Protein interaction networks were further investigated for overrepresented pathways using the Reactome analysis tool [37]. The CRISPR/Cas9 activation screen was performed *n* = 1.

### Generation of clonal cell lines overexpressing NAE1

Production of lentiviral particles was performed in HEK293T cells using two helper plasmids (pMD2.G, psPAX2) and the pLV-NAE1-GFPSpark plasmid encoding a NAE1/GFP fusion protein (Sino Biological, Beijing, China). For virus concentration, the Lenti-X-concentrator (Takara BIO INC.) was applied according to the manufacturer’s protocol. JAR and 2102EP cells were transduced with 50x concentrated lentivirus and expanded for 6 days. For the generation of 2102EP^NAE1/GFP^ and JAR^NAE1/GFP^ clonal cell lines, single cell FACS sorting was performed using the BD FACS ARIA III/BD FACS Melody (BD Biosciences, East Rutherford, NJ, USA). To discriminate dead cells DAPI (AppliChem GmbH, Darmstadt, Germany) staining was applied (5 µg/ml). GFP (NAE1) positive, DAPI-negative (alive) cells were collected in 96-well-cell culture plates and expanded.

### Protein extraction, SDS-PAGE and western blot

Protein extraction using RIPA buffer (Cell Signaling, USA), SDS-polyacrylamide gel electrophoresis (SDS-PAGE) and Western Blot analysis were performed as described previously [6, 31]. Information on primary and secondary antibodies used are provided in Supplementary Table S3.

### Cell viability assay

After treatment of TGCT cells with MLN4924 (solvent dimethylsulfoxid (DMSO)), cisplatin (solvent dimethylformamide (DMF)) or a combination of both drugs, XTT viability assay was performed as described [38]. Means of technical triplicates were calculated and related to solvent control (DMSO, DMF, combination). This procedure was repeated three times resulting in three independent biological replicates (*n* = 3). Viability assays of NAE1 overexpression cell lines treated with cisplatin or solvent were performed *n* = 4.

### DAPI/AnnexinV (apoptosis) and Hoechst-FACS analysis (cell cycle)

One day after seeding in 6-well cell culture plates (1.5 × 10^5^ cells/well), treatment with MLN4924, cisplatin, combination of both drugs or solvent (DMSO, DMF or DMSO/DMF combination) was performed. Subsequent FACS-based apoptosis (DAPI/PE AnnexinV) and cell cycle analysis was applied as described (*n* = 3) [31]. For apoptosis analysis, combination treatment was compared to MLN4924 or cisplatin-only application while for cell cycle analysis, the cell cycle phases of the drug-treated groups were compared to the solvent-treated controls (*n* = 3).

### 3’mRNA sequencing analysis

JAR and 2102EP cells were seeded into six-well cell culture plates (10^5^ cells/well). The next day, MLN4924 (JAR 1 µM, 2102EP 0.25 µM), cisplatin (JAR 4 µM, 2102EP 2.5 µM) and combination treatment (JAR 1 µM MLN4924 and 4 µM CP, 2102EP 0.25 µM MLN4924 and 2.5 µM CP) was started. After 2 days, RNA was isolated using RNeasy Mini Kit (Qiagen, Hilden, Germany). RNA quality control, library preparation, 3’mRNA sequencing and bioinformatic evaluation were done by the Next Generation Sequencing Core Facility (Bonn University) as described [31]. Differential expression data were investigated using STRING 11.5 database [36], integrated Gene Ontology [39, 40] and Reactome [37] analysis tools. For each cell line and condition *n* = 3.

### Statistical analysis

Independent biological replicates are displayed as mean ± SD (standard deviation) given as error bars. For statistical comparison of two groups, two-tailed Student’s *t* test was applied. Datapoints with significant difference (*P* < 0.05) were indicated with asterisks.

## Results

### Application of genome-scale CRISPR/Cas9 activation screen yields cisplatin-resistant TGCT cells

A saturating CRISPR/Cas9 SAMv2 transcriptional activation screen was applied to generate cisplatin-resistant JAR^MPHv2/SAMv2^/2102EP^MPHv2/SAMv2^ cells as outlined in Fig. 1b and Supplementary Fig. S1A. The SAMv2 plasmid library was amplified, purified and analysed by next-generation sequencing (NGS) revealing a coverage of more than 99.9% of sgRNAs and a skew ratio below 6 indicating equal distribution of the sgRNAs, important for maximum screening efficiency (Supplementary Fig. S2A, B). To reach a sgRNA coverage of 100% >1.1 × 10^8^ JAR^MPHv2^ and 2102EP^MPHv2^ cells were transduced with the SAMv2 library. Next, cisplatin treatment was started and cytotoxic effects were monitored (Supplementary Fig. S1B, C). Cell density decreased in the gene-edited cells as well as in the control cells. However, during the recovery phase in medium without cisplatin several gene-edited cells restarted proliferation, indicating that SAM-mediated activation of target genes had induced a selective advantage, the control cells died. To confirm cisplatin resistance, surviving cells were re-treated with cisplatin. Bright-field images of the gene-edited cells portended a higher viability compared to the treated control indicated by higher cell number, suggestive of cisplatin resistance (Supplementary Fig. S3A, B).

To identify genes responsible for cisplatin resistance, the integrated sgRNA regions were analysed using NGS. All candidate genes with a read count >10,000 are depicted in Fig. 1c. A complete list is provided in Supplementary Fig. S4 and Supplementary Data Set S1. Interestingly, when comparing candidate genes identified in 2102EP^MPHv2/SAMv2^ and JAR^MPHv2/SAMv2^, only 3 out of 93 genes overlapped, suggesting cell line/tumour type-specific mechanisms of cisplatin resistance (Fig. 1d). STRING interaction analysis of JAR^MPHv2/SAMv2^ candidate genes revealed a network including *TRAP1*, *CDC37*, *DDB1*, *NAE1*, *POLE4*, *NUP133*, *SGOL2* and *TK1*. Reactome database analysis performed on this network resulted in significantly enriched terms associated with cell cycle, DNA repair and neddylation (Fig. 1e). For 2102EP^MPHv2/SAMv2^, three candidate genes (*TK1*, *DDB1* and *AKT3*) were found to be part of these pathways. Since disturbed cell cycle and DNA repair are already known in the context of cisplatin resistance in TGCTs [41], we decided to investigate the role of NAE1-mediated neddylation.

### Overexpression of NAE1 results in cisplatin resistance

**Fig. 2:**
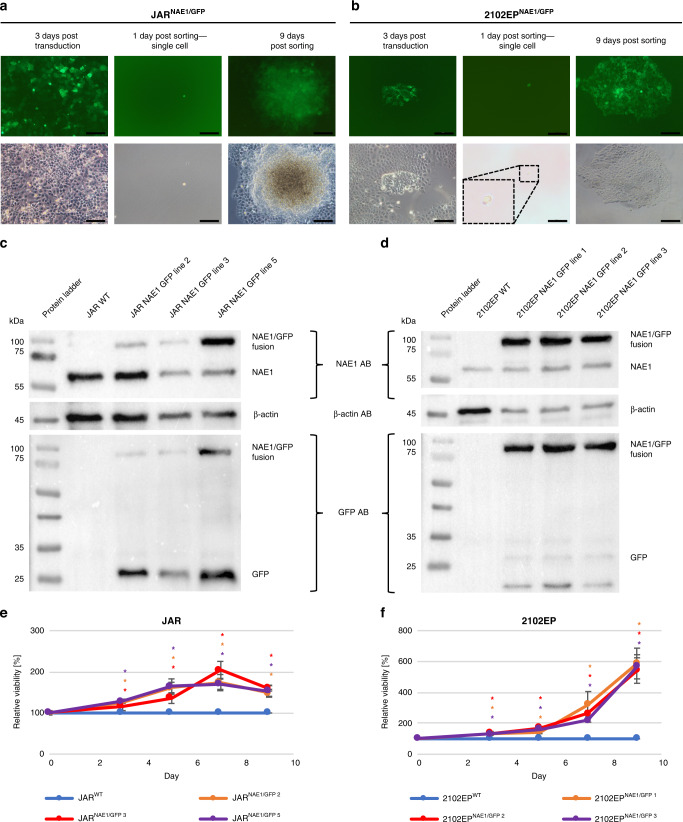
Generation, validation and analysis of clonal cell lines overexpressing NAE1/GFP. Cells were transduced with NAE1/GFP lentiviral particles. FACS sorting collected GFP-positive and DAPI-negative cells. Clonal (**a**) JAR^NAE1/GFP^ and **b** 2102EP^NAE1/GFP^ cell lines were cultured from single cells. Fluorescent and bright-field pictures are shown (scale bar 100 μm). **c**, **d** Western Blot analysis detecting GFP, NAE1 and β-actin (loading control) in JAR and 2102EP cells. **e** JAR^NAE1/GFP^ 2, 3, 5 and JAR WT as well as (**f**) 2102EP^NAE1/GFP^ 1, 2, 3 and 2102EP WT cell lines were treated with 5 and 2.5 μM cisplatin, respectively. Next, viability was measured via XTT assay at days indicated and was normalised to DMSO control which was set to 100% and to the wild-type cell line at the respective day. Asterisks indicate a significant difference between NAE1/GFP cell lines and WT line at each time point (*P < 0.05). *n* = 4. Data represent independent biological replicates mean ± SD.

To confirm the results from the genome-scale screen, clonal JAR^NAE1/GFP^ and 2102EP^NAE1/GFP^ cell lines overexpressing *NAE1* were generated. A CMV-driven NAE1-GFP fusion was transduced (Fig. 2a, b), and protein level was analysed via western blot (Fig. 2c, d). The band at 87 kDa was detected with both, NAE1- and GFP-antibody suggesting a NAE1-GFP fusion protein. Wild-type NAE1 was detected at 60 kDa. Further, an additional GFP band at 27 kDa is suggestive of cleavage of the protein.

Next, the 2102EP^NAE1/GFP^ and JAR^NAE1/GFP^ cells were tested for cisplatin resistance (Fig. 2e, f) showing a significant higher viability upon cisplatin treatment compared to wild-type cells (2102EP^NAE1/GFP^ 5.5-fold; JAR^NAE1/GFP^ 1.5-fold).

### Neddylation inhibitor MLN4924 in combination with cisplatin effectively reduces cell viability in TGCT cell lines

**Fig. 3:**
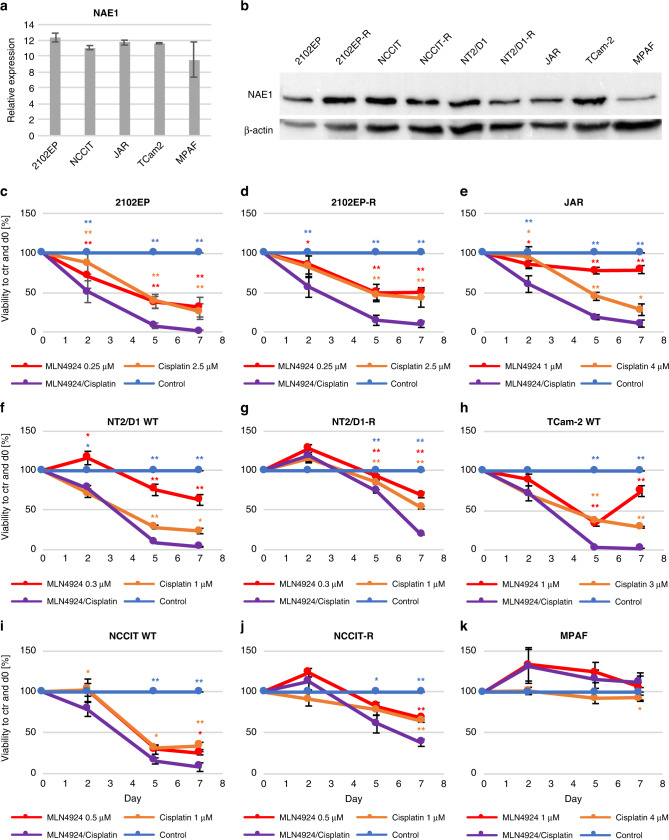
Effect of NAE1 inhibitor MLN4924, cisplatin or MLN4924/cisplatin treatment on wild-type and cisplatin-resistant TGCT cell lines. **a** Meta-analysis of Illumina microarray data from TGCT cell lines (2102EP, NCCIT, JAR, TCam-2) and fibroblast cells (MPAF) for *NAE1* mRNA expression levels. **b** Western Blot analysis of NAE1 protein levels in TGCT cell lines and control cells with corresponding β-actin loading control. Viability of (**c**) 2102EP, (**d**) 2102EP-R, (**e**) JAR, (**f**) NT2/D1, (**g**) NT2/D1-R, (**h**) TCam-2, (**i**) NCCIT, (**j**) NCCIT-R and (**k**) MPAF cells was analysed. Treatment was repeated and XTT assay was performed at days indicated. All values were normalised to the corresponding solvent control (100%) and referred to day 0. Asterisks indicate significant change in viability between any condition compared to the combination treatment (*P < 0.05). Asterisks colour code indicate the corresponding treatment condition. *n* = 3. Data represent independent biological replicates mean ± SD.

If overexpression leads to cisplatin resistance, then inhibition of neddylation should result in cisplatin sensitivity. We performed *NAE1* gene expression meta-analysis of microarray data on different cell lines and tissues previously published by us [42, 43]. TGCT cell lines as well as TGCT tissues revealed high levels of *NAE1* mRNA (Fig. 3a and Supplementary Fig. S5A) while expression was lower in MPAF cells and normal testes tissue. Further, Western Blot analysis demonstrated high NAE1 protein levels for 2102EP, 2102EP-R, NCCIT, NCCIT-R, NT2/D1, NT2/D1-R, JAR and TCam-2 cell lines (Fig. 3b). MPAF cells revealed lower levels of the target protein. Further, using Firebrowse database analysis higher *NAE1* expression in TGCTs compared to other tumour entities were found (Supplementary Fig. S5C). UCSC Xena browser-mediated analysis of NAE1 isoforms in TGCT tissue compared to normal testis tissue showed differential expression of several isoforms (Supplementary Fig. S5B). Next, we performed Western blot analysis revealing increased protein levels of p27 in 2102EP, JAR and TCam-2 cell lines (Supplementary Fig. S6A–C) treated with MLN4924, pointing towards suppression of neddylation [24, 44]. On the same blots, we detected an accumulation of γH2A.X after cisplatin treatment indicating DNA damage induction [45, 46]. Consequently, increased p27 and γH2A.X levels were also found in the cells treated with MLN4924/cisplatin combination suggesting inhibition of neddylation and induction of DNA damage, which might trigger a stronger cytotoxic effect compared to monotherapy.

Next, we performed XTT viability assays after treatment of 2102EP, 2102EP-R, NCCIT, NCCIT-R, NT2/D1, NT2/D1-R, JAR and TCam-2 cell lines as well as MPAF cells (Fig. 3c–k). For all TGCT cell lines, a decrease in viability was observed starting latest from day 5 of treatment with MLN4924, cisplatin or MLN4924/cisplatin compared to solvent controls. The combination treatment had a strong additive effect, which was evident by significantly reduced viability (viability at day 7 referred to the control: 2102EP 1%, NT2/D1 4%, NCCIT 8%, JAR 11%, TCam-2 2%) compared to the single applications (MLN4924 only/cisplatin only at day 7: 2102EP 32/26%, NT2/D1 63/24%, NCCIT 25/34%, JAR 79/28%, TCam-2 75/29%) and to the control (100%). Strikingly, even in the cisplatin-resistant 2102EP-R, NCCIT-R and NT2/D1-R cell lines a significantly lower viability for combination treatment was observed (MLN4924 only/ cisplatin only/combination at day 7: 2102EP-R 50/43/10%, NT2/D1-R 70/54/19%, NCCIT-R 69/65/38%). The MPAF control cells did not show any decrease in viability in all applied drug concentrations. Our findings revealed a strong reduction in viability after MLN4924, cisplatin and MLN4924 in combination with cisplatin treatment in all TGCT cell lines including seminoma-, EC-, CC-cell lines and cisplatin-resistant sub cell lines. Importantly, combination resulted in further reduction of viability.

### MLN4924 and cisplatin induce apoptosis and cell cycle arrest

**Fig. 4:**
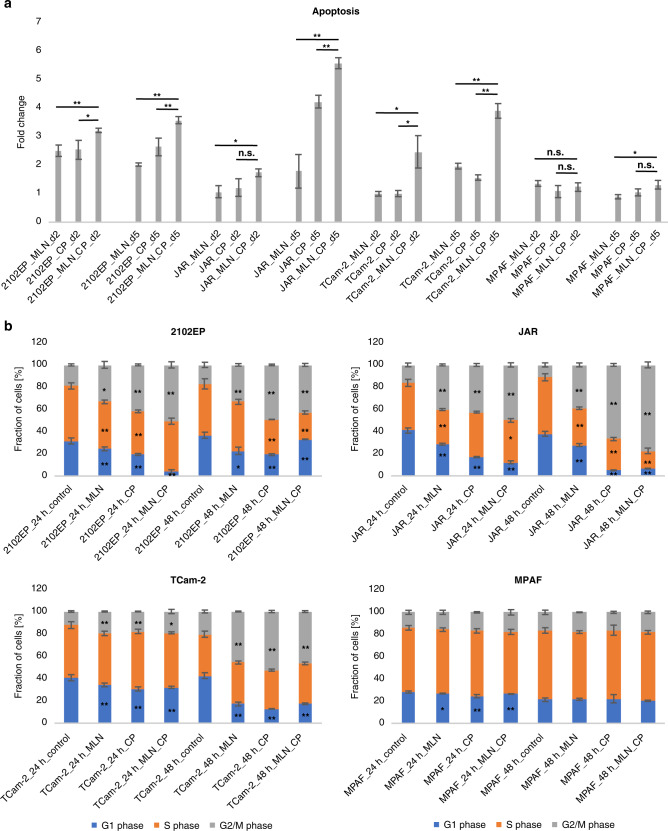
NAE1 inhibition and cisplatin treatment induce apoptosis and cell cycle arrest in TGCT cell lines. **a** DAPI/Annexin-V FACS-based apoptosis analysis was performed after treatment of the cells with MLN4924 only (2102EP 0.25 μM, TCam-2, JAR and MPAF 1 μM), cisplatin only (2102EP 2.5 μM, TCam-2 3 μM, JAR and MPAF 4 μM) or MLN4924/cisplatin combination (same as MLN4924 and cisplatin only) for 2 and 5 days. Drug application was repeated after 2 days. Treated samples were normalised to the solvent control referred to as fold change 1. Asterisks indicate significant difference in apoptosis between MLN4924 only or cisplatin only compared to the combination treatment (n.s. not significant, *P < 0.05, **P < 0.01). (*n* = 3). **b** For Hoechst-FACS-based cell cycle analysis cells were measured after 24 and 48 h of treatment (same concentrations as indicated above). Solvent-treated cells served as control sample. Asterisks indicate significant difference of cell portion in the respective cell cycle phase after treatment compared to control sample (*P < 0.05, **P < 0.01). (*n* = 3) Data represent independent biological replicates mean ± SD.

For further investigation of cellular effects, we focused on 2102EP, JAR, TCam-2 and MPAF cells. Apoptosis was analysed by FACS after 2 and 5 days of MLN4924, cisplatin or combination treatment (Fig. 4a). In 2102EP cells induction of apoptosis could be observed already after 2 days of treatment. Apoptosis induction at day 2 was 3.2 times stronger upon co-treatment, increasing to 3.6 times at 5 days of treatment. A strong increase in apoptosis from 2 to 5 days of combination treatment was also detected in JAR (1.7–5.6 times fold change) and TCam-2 (2.5–3.9 times fold change). The number of apoptotic cells was significantly increased in 2102EP (2 and 5 days), JAR (5 days) and TCam-2 (2 and 5 days) after combination treatment compared to application of MLN4924 or cisplatin monotreatment. MPAF cells showed almost no apoptosis induction.

Further, cell cycle analysis after 24 and 48 h of treatment was performed. Disturbance of the cell cycle was already detected in 2102EP cells after 24 h of treatment (Fig. 4b). The fraction of cells in G2/M-phase significantly increased while cell number in G1-phase decreased, indicating G2/M-phase arrest after treatment with MLN4924, cisplatin or both. The G2/M-phase cell cycle arrest persisted and could be detected after 48 h for all different conditions. After 24 h of treatment JAR cells accumulated in G2/M-phase which became even more striking after 48 h of treatment. For the JAR cells disturbance of the cell cycle appeared more severe after combination treatment compared to MLN4924 or cisplatin alone. TCam-2 cells showed only slight changes in cell cycle distribution after 24 h of drug application, but significant G2/M-phase arrest after 48 h. This is most likely due to higher doubling time of TCam-2 compared to 2102EP and JAR cells. MPAF cells treated with the highest concentration of MLN4924 and cisplatin applied here exhibited only minor changes of cell distribution in the cell cycle, indicating that the control cells are affected only to a very low extent.

### Transcriptome analysis revealed apoptosis induction, cell cycle arrest and cell differentiation after MLN4924/cisplatin combination treatment

To further investigate the molecular mechanisms of MLN4924 and cisplatin treatment in TGCTs, the transcriptome of 2102EP as well as JAR cells was analysed by 3’mRNA sequencing after 2 days of treatment (Supplementary Data Set S1).

**Fig. 5:**
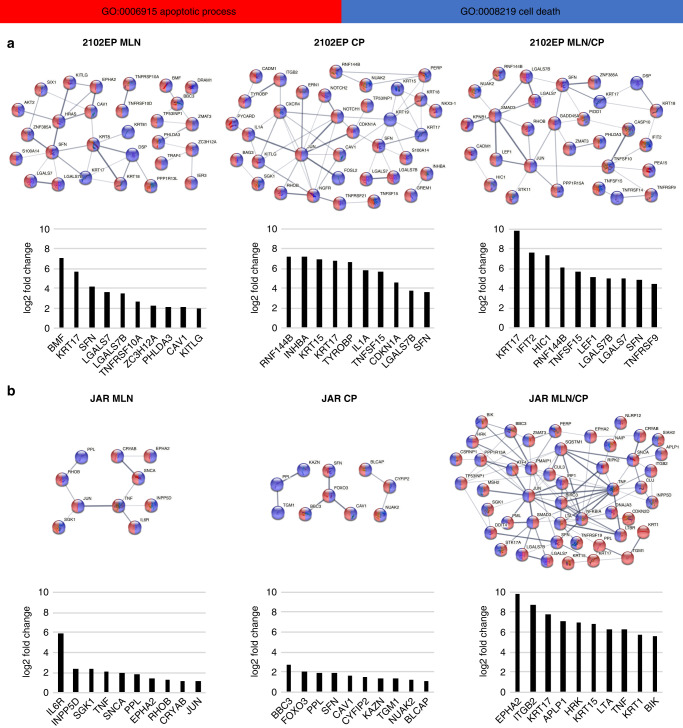
Apoptosis induction and disturbed cell cycle on transcriptome level after neddylation inhibition and cisplatin treatment. 2102EP/JAR cells were treated for 2 days with MLN4924 (0.25/1 μM), cisplatin (2.5/4 μM) or combination of both drugs. Gene ontology and STRING interaction analysis of differentially upregulated genes revealed significantly enriched (**a**) apoptosis and (**b**) cell cycle-related terms and networks. Log2 fold change is shown for top ten differentially upregulated genes of each network. For all samples *n* = 3. Data represent independent biological replicates mean.

STRING interaction analysis of differentially upregulated genes in 2102EP and JAR cells after MLN4924, cisplatin and combination treatment revealed interaction networks which were associated with the GO terms apoptosis and cell death (Fig. 5a, b and Supplementary Fig. S7A–F). The data suggested apoptosis induction after single drug and combination therapy, confirming the results from the FACS analysis. Further, based on the fold change of the top 10 upregulated genes in the network of 2102EP cells, apoptosis initiation appeared stronger upon combination treatment. Comparing the transcript levels of *KRT17*, an important player in apoptosis, showed a log2 fold change of 5.8/6.8/9.9 after MLN4924/cisplatin/combination treatment, respectively. In the JAR cell line, the effect of combination treatment was even more obvious. Here, the interaction network for MLN4924/cisplatin includes 48 proteins, while for MLN4924 only and cisplatin only there were just 10 proteins found for each condition associated with apoptosis and cell death. The increased apoptotic effect due to combination treatment can be explained when comparing the top ten upregulated genes in the interaction network, which showed strongly increased transcript abundancy compared to MLN4924 or cisplatin-only application.

Further, transcriptome analysis revealed cell cycle arrest, indicated by comprehensive STRING networks for 2102EP cells treated with MLN4924 and combination treatment (Supplementary Fig. S8A–F). MLN4924 treated 2102EP cells revealed a complex network with differentially downregulated genes like *CCNA1, TUBB4B, KIFC1, BUB3* suggesting downregulation of cell cycle progression. In contrast, cisplatin application in 2102EP cells resulted only in a small network of upregulated genes associated with cell cycle arrest, including the cell cycle regulator p21 (*CDKN1A*). Combination treatment revealed a network of differentially upregulated genes involved in negative regulation of cell cycle, like *SFN*, *GPER*, etc. In JAR cells, many differentially downregulated genes associated with cell cycle progression were found (Supplementary Fig. S9A–F). Especially after combination treatment, the results tended towards decreased cell cycle processes (*FES, HSF1, PIN1*), while after MLN4924 or cisplatin application only few cell cycle-related transcripts were differentially expressed.

**Fig. 6:**
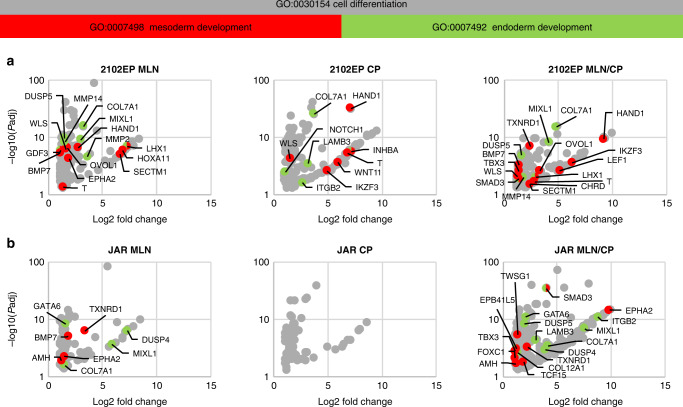
NAE1 inhibition and cisplatin treatment drive the cells into differentiation. Investigation of the transcriptome by 3’mRNA sequencing and subsequent Gene Ontology analysis. Dot blots indicate log2 fold change and –log10 *P* adjusted value of transcripts which were higher abundant after treatment compared to control. Cell differentiation-associated transcripts are depicted as grey dots while transcripts associated with mesoderm as well as endoderm development are highlighted in red and green, respectively. 3’mRNA sequencing of (**a**) 2102EP and **b** JAR cells was performed 2 days after application of MLN4924 (0.25/1 μM), cisplatin (2.5/4 μM) or combination of both drugs. For all samples *n* = 3. Data represent independent biological replicates mean.

Analysis of 3’mRNA sequencing data after two days of treatment with MLN4924, cisplatin or combination revealed initiation of cellular differentiation in 2102EP and JAR cells (Fig. 6a, b and Supplementary Fig. S10A–F). We found highly increased transcript abundance of cell differentiation markers, like *HAND1*, *CDX2*, *CLDN1* and *SOX15* after all three treatment conditions in 2102EP cells. Co-treatment of JAR cells also revealed strong upregulation of *CDX2* and *SOX15*. Especially, the combination treatment seemed to drive the 2102EP and JAR cells into mesoderm and endoderm fate, which was supported by differential upregulation of marker genes like *MIXL1, COL7A1, SMAD3, TXNRD1, DUSP5* and *TBX3*. In parallel to the induction of differentiation, pluripotency markers, such as SOX2, *SOX21*, *KLF15*, *TCFL1* and *HESX1* (only deregulated in MLN4924/cisplatin sample) were strongly downregulated in 2102EP cells treated with MLN4924 only or MLN4924 in combination with cisplatin (Supplementary Fig. S11A–D).

## Discussion

Here, we used a genome-scale CRISPR/Cas9 activation screen to uncover pathways contributing to cisplatin resistance in TGCTs. Among known genes involved in DNA repair and cell cycle control, we identified components of the neddylation pathway to mediate resistance. Overexpression of *NAE1* in 2102EP and JAR cells resulted in increased cisplatin resistance, validating our finding. In turn, inhibition of neddylation increased cisplatin sensitivity of TGCT cell lines. The combination treatment of the covalent NAE1 inhibitor MLN4924 and cisplatin revealed a highly therapeutic efficacy, not only in the tested TGCT parental cell lines, but also in the cisplatin-resistant lines. Further, we found that the fibroblast control cells were unaffected by the combination therapy, suggesting no severe side effects. Next, we could show that MLN4924 in combination with cisplatin resulted in stronger G2/M-phase cell cycle arrest and increased apoptosis induction compared to monotherapy. In 2102EP, JAR and TCam-2 cells on protein level strong accumulation of p27 after MLN4924 only and MLN4924/cisplatin treatment was found. Further, accumulation of γH2A.X after cisplatin and MLN4924/cisplatin treatment was observed validating known targets of MLN4924 and cisplatin. Transcriptome analysis in 2102EP and JAR cells revealed strong induction of meso- and endoderm fate, which was most prominent after combination treatment. In 2102EP cells, downregulation of pluripotency markers was observable after MLN4924 only and MLN4924/cisplatin combination treatment.

The neddylation core component NAE1 was the most interesting factor we found in the genome-scale CRISPR/Cas9 activation screen. Surprisingly, there is nothing known about the role of neddylation in TGCTs. Overactivation of neddylation in different tumour entities like liver, lung and colon cancer suggest an important role in carcinogenesis. In pancreatic cancer overexpression of *NAE1* has even been shown to be responsible for cisplatin resistance [27]. Further, we found overactivated *DDB1* in cisplatin-resistant TGCT cells which is aside from DNA repair also involved in the neddylation cascade. In detail, DDB1 is part of the CRL4 complex which in cisplatin-resistant ovarian cancer seems to be considerably overactivated. There cisplatin resistance could be overcome by knockdown of CLR4 [47]. Here, we suggest overactivated NAE1/CLR4 axis to represent a putative pathway for cisplatin resistance. In this study, we could already proof that overexpression of *NAE1* in 2102EP^NAE1/GFP^ and JAR^NAE1/GFP^ cells induced cisplatin resistance which makes neddylation a very interesting and promising therapeutic target in treatment of TGCTs.

The genome-scale CRISPR/Cas9 activation screen we applied in our study also revealed already known cisplatin resistance factors, like altered cell cycle and disturbed DNA repair mechanisms in TGCTs [14, 15]. In cisplatin-resistant JAR^MPHv2/SAMv2^ and 2102EP ^MPHv2/SAMv2^ cells, we found *POLE4* as well as *AKT3* to be upregulated, respectively. Both proteins have already been described as factors contributing to cisplatin resistance in TGCTs [14, 15, 48, 49]. POLE4 is a subunit of DNA polymerase ε playing a role in nucleotide excision repair, base excision repair, DNA double-strand break repair and proofreading, supporting genome stability [48, 49]. High levels of AKT3 induce overactivation of the PI3K/AKT signalling pathway preventing cell cycle arrest and apoptosis [14, 15]. These results clearly validate the CRISPR screen since we found not only novel but also already known factors of cisplatin resistance in TGCTs.

Further, in cisplatin-resistant cells from our screen upregulated genes like *TRAP1*, *TK1* and *SGOL2* were identified. Elevated expression level of the mitochondrial heat shock protein TRAP1 is associated with cisplatin resistance in osteosarcoma, colorectal, prostate and lung cancer due to reduction of reactive oxygen species production and prevention of apoptosis. Knockdown of TRAP1 increased sensitivity toward cisplatin [50, 51]. Thus, TRAP1 could also be an interesting prognostic indicator and therapeutic target for cisplatin-resistant TGCTs. *TK1* and *SGOL2* have been identified as tumour progression factors in other tumour entities [52, 53]. Increased TK1 protein levels were found to serve as a biomarker for poor prognosis after initial chemotherapy of lung, ovarian and breast cancer [52]. *SGOL2* is defined as an oncogene in hepatocellular carcinoma cells [53]. We hypothesize that upregulation of *TK1* and *SGOL2* promotes cell proliferation in TGCTs thereby initiating cisplatin resistance which makes these genes interesting candidates for further investigations.

The genome-scale CRISPR/Cas9 screen applied here revealed already known and novel factors responsible for cisplatin resistance in TGCTs. Since the screen was performed *n* = 1 we do not claim to display a comprehensive list of all possible cisplatin resistance factors in TGCTs. Instead, this study revealed several interesting candidate genes which might contribute to cisplatin resistance in TGCTs. The screen can only deliver initial suggestions which have to be confirmed as shown for *NAE1*.

Next, treating TGCT cells with both MLN4924 and cisplatin revealed an additive cytotoxic effect displayed by decreased viability. Of note, MLN4924/cisplatin combination treatment in a xenograft mouse model of pancreatic cancer resulted in significantly decreased tumour volume [27]. Further, co-treatment of the TGCT cell lines led to G2/M-phase cell cycle arrest and apoptosis induction. Application of both drugs also resulted in protein accumulation of p27 and γH2A.X indicating the additional effect of combination treatment. In other tumour entities like lung cancer and oesophageal squamous cell carcinoma MLN4924 has been shown to induce G2/M-phase cell cycle arrest [25, 54]. Both publications describe the accumulation of p21, p27 and Wee1 after MLN4924 treatment as a cause for cell cycle arrest and observed subsequent apoptosis [25, 54]. Cisplatin-based apoptosis follows a similar pattern, including DNA damage indicated by increased γH2A.X levels, subsequent G2/M-phase cell cycle arrest and Caspase as well as PUMA and NOXA-mediated apoptosis [41, 48]. Thus, our findings are in accordance with the literature revealing MLN4924 and cisplatin combination treatment leads to enhanced G2/M-phase cell cycle arrest and subsequent apoptosis induction in TGCTs.

Interestingly, in 2102EP as well as in JAR cells, after MLN4924, cisplatin and combination treatment we found mRNA levels of *KRT17* to be highly upregulated. According to the gene ontology analysis *KRT17* is involved in apoptosis induction. In pancreatic cancer overexpression of *KRT17* was shown to decrease CyclinD1 levels and increase cleaved caspase 3 amounts causing cell cycle arrest and apoptosis [55]. Thus, upregulation of *KRT17* in JAR and 2102EP cells might contribute to induction of apoptosis.

We observed reduced transcript levels of *SOX21* and of the core EC marker *SOX2* after MLN4924 only and cisplatin/MLN4924 combination treatment of 2102EP cells, suggesting downregulation of pluripotency. At the same time in 2102EP as well as in JAR cells treated with both drugs strong induction of cell differentiation was found. Especially mRNA levels of *CDX2* and *HAND1* were highly upregulated in 2102EP cells after treatment whereas in JAR cells a moderate upregulation has been detected. Concomitant downregulation of *SOX2* and *SOX21* while maintaining CDX2 levels induces cell differentiation [56]. In 2102EP mesoderm fate is induced indicated by upregulation of 13 mesoderm-associated genes including *HAND1*, *LEF1*, etc. JQ1 mediated inhibition of BRD2/4 results in downregulation of pluripotency and induction of cell differentiation (mesoderm development) in NCCIT cells [6]. Differentiation of tumour cells can be advantageous by limiting their progression and metastatic potential. Especially ECs show reduced tumorigenicity due to treatment with differentiation agents like thioridazine and salinomycin [57]. Surprisingly, MLN4924 treatment of JAR cells resulted in upregulation of four mesoderm- (*TXNRD1*, *BMP7*, etc.) and four endoderm-associated (*DUSP4*, *MIXL1*, etc.) genes. Of note, JAR is a cell line derived from choriocarcinoma and displays extraembryonic characteristics. Due to the absence of upregulation of EC markers we speculate that MLN4924 induces a direct reprogramming from extraembryonic to meso- and endoderm fate. Since we previously demonstrated reprogramming from seminoma to EC-like fate and from seminoma to mixed-non-seminoma fate [9, 10, 58], conversion of JAR cells reported here further underscores the inherent plasticity of TGCTs.

*CDX2* overexpression can be indicative for differentiation into YST and Ter. Further, we found upregulation of *GATA3* and *KRT17* in 2102EP cells after combination treatment. Increased GATA3 levels were detected in several YST patients but seem to be more common in CC [59]. Elevated *KRT17* expression rather points towards Ter differentiation [60]. However, since the key markers α-Fetoprotein, Glypican-3, β-HCG are not expressed differentiation into YST, CC or Ter seems rather unlikely.

In the context of current pre-, on-, post- or off-target effect classification of cisplatin resistance, we consider global upregulation of neddylation as a pre- or a post-target effect. It remains elusive whether patients with resistant TGCTs display already high levels of NAE1 or whether the treatment itself upregulates NAE1 levels. Increased neddylation of substrates such as cullins results in subsequent degradation of many cellular proteins, like tumour suppressors (p21, p27) and failure of apoptosis induction. In turn, inhibition of neddylation by MLN4924 results in accumulation of cell cycle inhibitors p21, p27, Wee1, etc. and factors for induction of apoptosis (e.g., NOXA accumulation) [24].

In other tumour entities an additive effect of MLN4924 in combination with cisplatin was observed at MLN4924 concentrations which were in the same range (0.2 to 1 µM) [27, 54] as in our study for the TGCTs. Here, MLN4924 treatment resulted in re-sensitisation of cisplatin-resistant cells. Interestingly, MLN4924 inhibitor in combination with cisplatin did not show any effect on the fibroblast control cells. NAE1 expression is highest in TGCTs compared to other tumour entities and normal tissues. According to The Human Protein Atlas and the Firebrowse database, in TCGT tissues neddylation pathway members are expressed at moderate to high levels. Thus, MLN4924 in combination with cisplatin appears as an attractive treatment option in TGCTs. At present, 40 clinical trials investigate the potential of MLN4924 in various tumour entities (https://www.clinicaltrials.gov/). This will allow an off-label use for patients suffering from TGCTs as soon as MLN4924 is approved.

To conclude, we identified upregulation of neddylation to contribute to cisplatin resistance in TGCTs. Combination treatment of NAE1 inhibitor MLN4924 and cisplatin elicits a strong additive cytotoxic effect in TGCTs while leaving fibroblast control cells unaffected. Further, inhibition of neddylation by MLN4924 effectively re-sensitised TGCT-R cells towards cisplatin. The fact that TGCTs display the highest levels of NAE1 compared to other human tumour entities or regular tissue suggest neddylation to be potential novel therapeutic option for TGCTs.

## Supplementary information

Supplementary Figures

Supplementary Methods

Supplementary Data Set

## Data Availability

Raw and processed 3’mRNA sequencing data generated in this study have been deposited in the Gene Expression Omnibus database under accession code GSE216043. The raw and processed DNA sequencing data (CRISPR/Cas9 screen) are available in the Gene Expression Omnibus database under accession code GSE225852. The Cancer Genome Atlas’ (TCGA) datasets can be accessed via the UCSC Xena browser (https://xena.ucsc.edu) and Firebrowse (https://www.http://firebrowse.org/). Protein expression datasets are available via The Human Protein Atlas (https://www.proteinatlas.org/).

## Code availability

The previously published algorithm count_spacers.py [32] was applied using Python 2.7 and Biopython 1.74.
