# Supplementary material for: Genome-scale CRISPR screen reveals neddylation to contribute to cisplatin resistance of testicular germ cell tumours
Source: Br J Cancer. 2023 Apr 6;128(12):2270–82. doi: 10.1038/s41416-023-02247-5 (PMC10241889; doi:10.1038/s41416-023-02247-5)
Supplement: Supplementary file 1 — Supplementary Figures [file 41416_2023_2247_MOESM1_ESM.pdf]

Genome-scale CRISPR screen reveals neddylation to contribute to cisplatin resistance of testicular germ cell tumors

-Supplementary Figures-

Kai Funke[1], Ulf Einsfelder[2], Aylin Hansen[1], Lena Arévalo[1], Simon Schneider[1], Daniel Nettersheim[3], Valentin Stein[2] and Hubert Schorle[1]

[1]Department of Developmental Pathology, Institute of Pathology, University Hospital Bonn, Bonn, Germany.

[2]Institute of Physiology II, University Hospital Bonn, Bonn, Germany

[3]Department of Urology, Urological Research Laboratory, Translational UroOncology, Medical Faculty and University Hospital Düsseldorf, Heinrich Heine University Düsseldorf, Düsseldorf, Germany

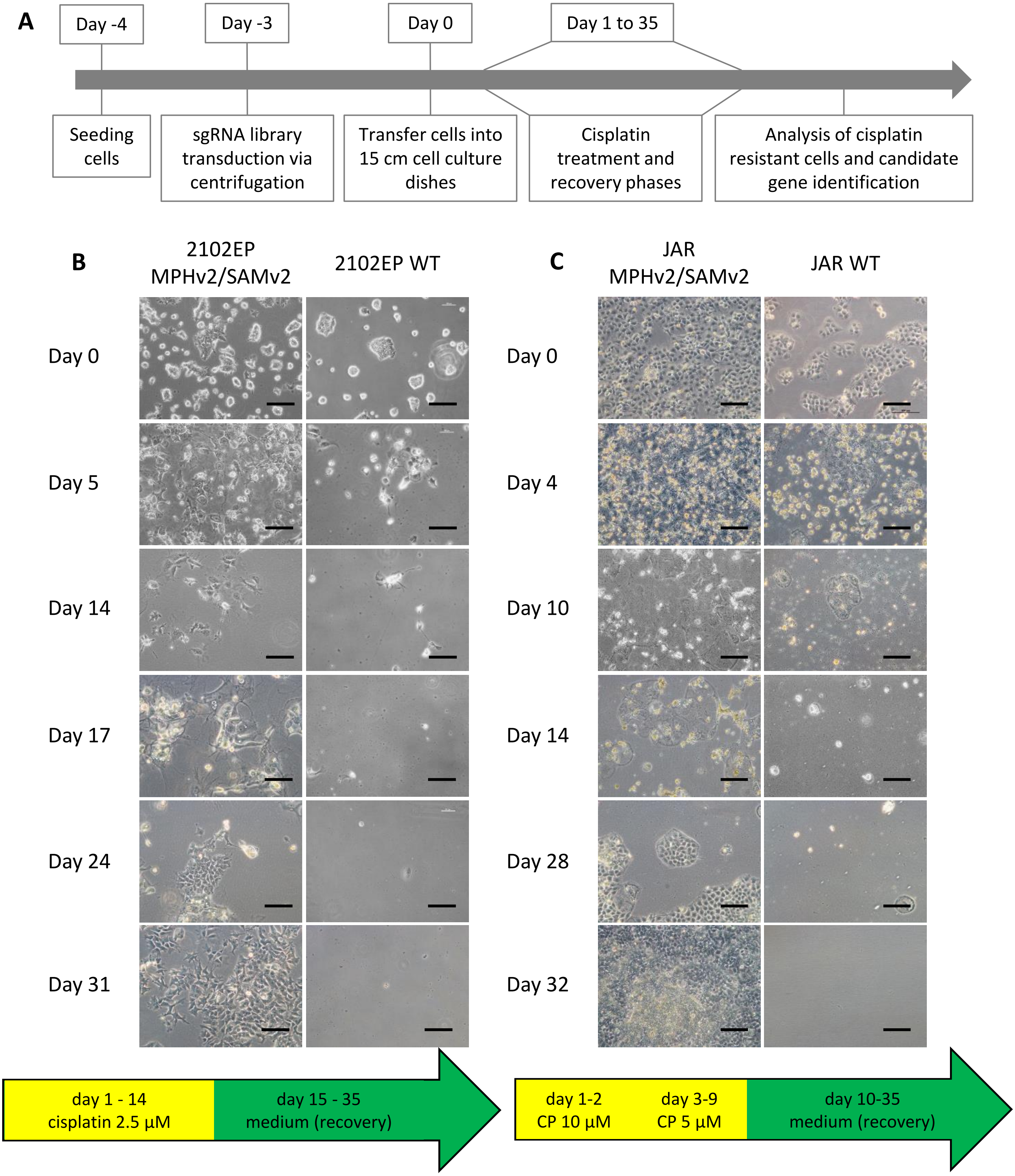

Supplementary Figure S1: Genome-scale CRISPR screen – cisplatin treatment and recovery. (A) Time line of genome-scale activation screen. After library transduction (B) 2102EP$^{MPHv2/SAMv2}$, (C) JAR$^{MPHv2/SAMv2}$ cells as well as corresponding wild type control were treated with different cisplatin regimens. Brightfield images highlight the effect of cisplatin and the difference between library transduced cells and wild type cells (scale bar: 100 µm). For 2102EP$^{MPHv2/SAMv2}$ and JAR$^{MPHv2/SAMv2}$ a sgRNA coverage of 99 to 100% was reached.

A

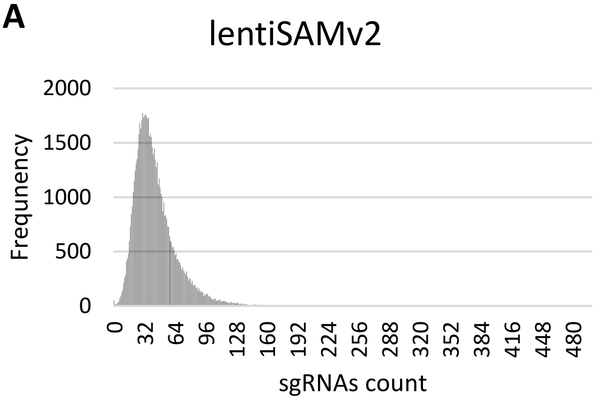

B

	lentiSAMv2
Detected sgRNAs [count]	70248
Detected sgRNAs [%]	99.93
Undetected sgRNAs [count]	49
Undetected sgRNAs [%]	0.07
Skew ratio of top 10% to bottom 10%	3.4
Guides total	70297
Reads total	3079894

Supplementary Figure S2: Genome-scale plasmid library – quality check. After library amplification and preparation next generation sequencing was performed to investigate library quality and sgRNA coverage. (A) Normal distribution of sgRNA frequencies and counts for lentiSAMv2 plasmid library. (B) Computational analysis was performed according to Joung *et al*. 2017.

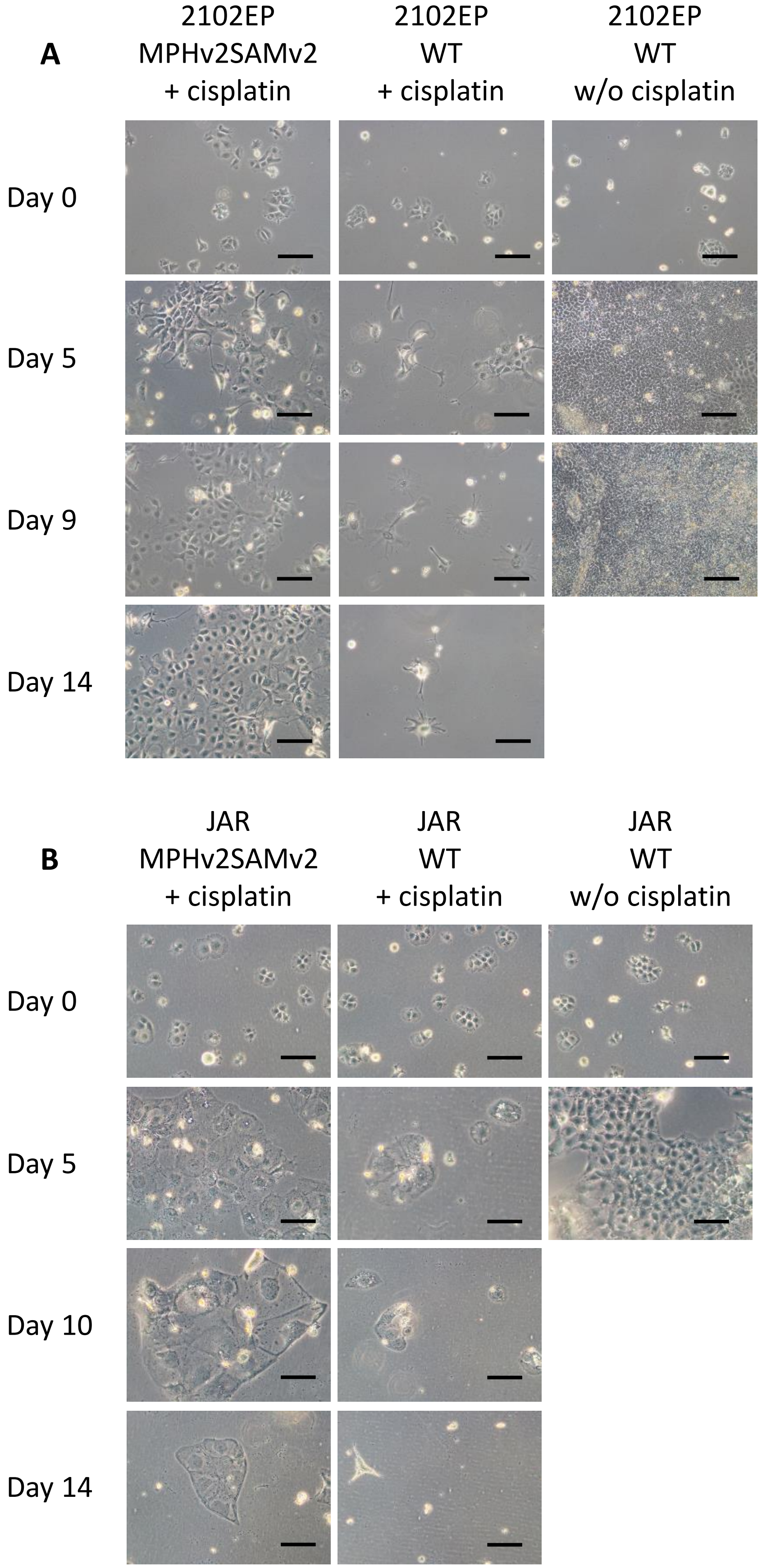

Supplementary Figure S3: Validation of cisplatin resistance by re-treatment. Surviving cells after initial genome-scale CRISPR/Cas9 based activation (SAMv2) screen were seeded again ($2.5x10^4$ cells per well of a 6 well cell culture plate) and re-treated with cisplatin. (A) $2102EP^{MPHv2/SAMv2}$, and $2102EP^{WT}$ cells (control) were seeded and treated with 2.5 µM cisplatin for 14 days. One additional wild type sample was kept in medium without cisplatin. (B) $JAR^{MPHv2/SAMv2}$ and JAR^{WT} cells (control) were seeded and treated with 5 µM cisplatin for 14 days. An additional wild type sample was cultured in medium without cisplatin. Scale bar: 100 µm.

Activation Screen (MPHv2SAMv2) – Candidate Genes										
2102 EP				JAR						
Rank	Gene name	read counts		Rank	Gene name	read counts		Rank	Gene name	read counts
1	TRAP1	8044032		1	CYP2R1	4248699		36	TRAP1	36686
2	TK1	7803883		2	LEKR1	4084952		37	OR8B3	36487
3	DDB1	6841409		3	CCL1	2230750		38	TK1	34429
4	ENSA	1590607		4	LHB	1223512		39	ARAF	30934
5	IFNW1	656648		5	WBP2NL	1066313		40	NAT14	30356
6	AKT3	531280		6	OR2W1	331952		41	NT5C3A	29223
7	PIR	499632		7	PLEKHF1	307083		42	KLK5	28747
8	AK1	496415		8	NAE1	179370		43	ING1	28599
9	KIF27	489821		9	CLDND2	171947		44	SGOL2	28571
10	GMNC	483723		10	ANGPTL1	167831		45	APOM	27737
11	CYP27A1	478347		11	CELF1	164345		46	DDB1	27734
12	PERP	462899		12	SOAT1	106411		47	CDC37	26990
13	MGAT4A	458608		13	AGPAT2	105646		48	SNRK	26934
14	ZC3H14	420023		14	EFR3B	95494		49	PAPD5	24778
15	CLEC2D	413240		15	TLCD1	94701		50	FBXO18	24447
16	DPP10	374926		16	KCNJ12	92110		51	NUP133	23166
17	C17orf103	359665		17	ALDH3B1	89330		52	ZNF676	20759
18	DDX10	295925		18	MDN1	87298		53	RHOBTB1	20123
19	KLHL36	230245		19	ALKBH3	81611		54	LOC441155	19573
20	SPDYC	224181		20	RNF165	76285		55	UNC79	19160
21	PP2D1	204439		21	CRY2	73850		56	SVOPL	18118
22	MTRNR2L6	186447		22	IRF2BP2	56826		57	OR6C1	17054
23	MTMR11	172159		23	CASC10	53587		58	LRRC43	16162
24	DNAJB6	149516		24	NPIPA7	53418		59	GPD2	13226
25	GCC1	125790		25	LEPROTL1	53049		60	EEF2K	13037
26	FAM115A	124352		26	CALCOCO2	50726		61	TGIF1	12622
				27	SCAND1	49872		62	BMF	12391
				28	RUNX1	48367		63	IGSF10	12082
				29	FADS1	45803		64	PHGR1	12035
				30	POLE4	43866		65	DHRS12	11884
				31	CPT1C	42620		66	C1orf189	11776
				32	ITSN1	42605		67	CEP350	11669
				33	ANK1	40835		68	LDLRAD4	11262
				34	HTR3E	40078		69	HESX1	11134
				35	KRT71	36909		70	USP6NL	10717

Supplementary Figure S4: Full list of candidate genes based on the genome-scale CRISPR/Cas9 activation screen (MPHv2/SAMv2). Candidate genes with a threshold of read count higher than 10000 are shown for 2102EP and JAR cells. Bioinformatic analysis of the NGS results was performed using Biopython.

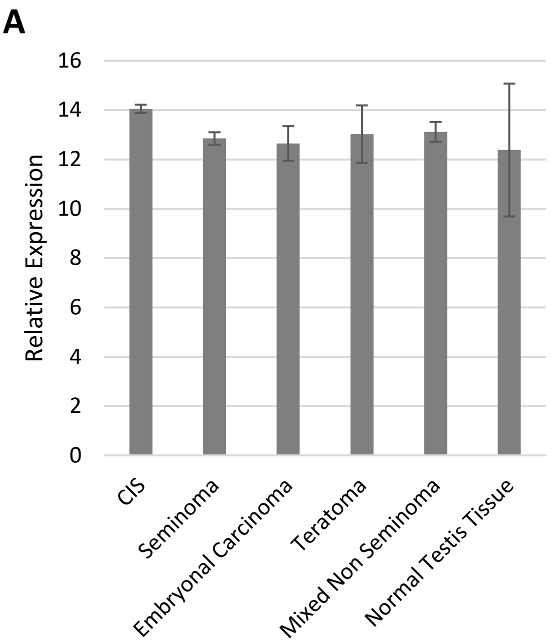

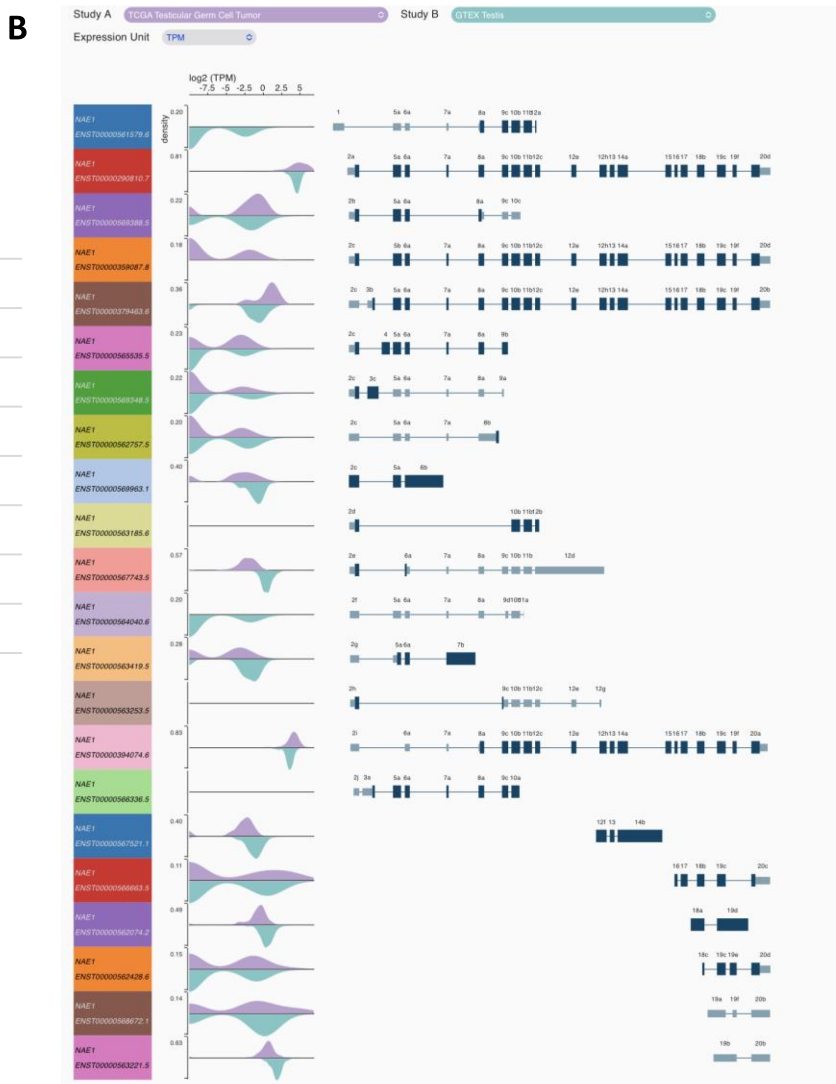

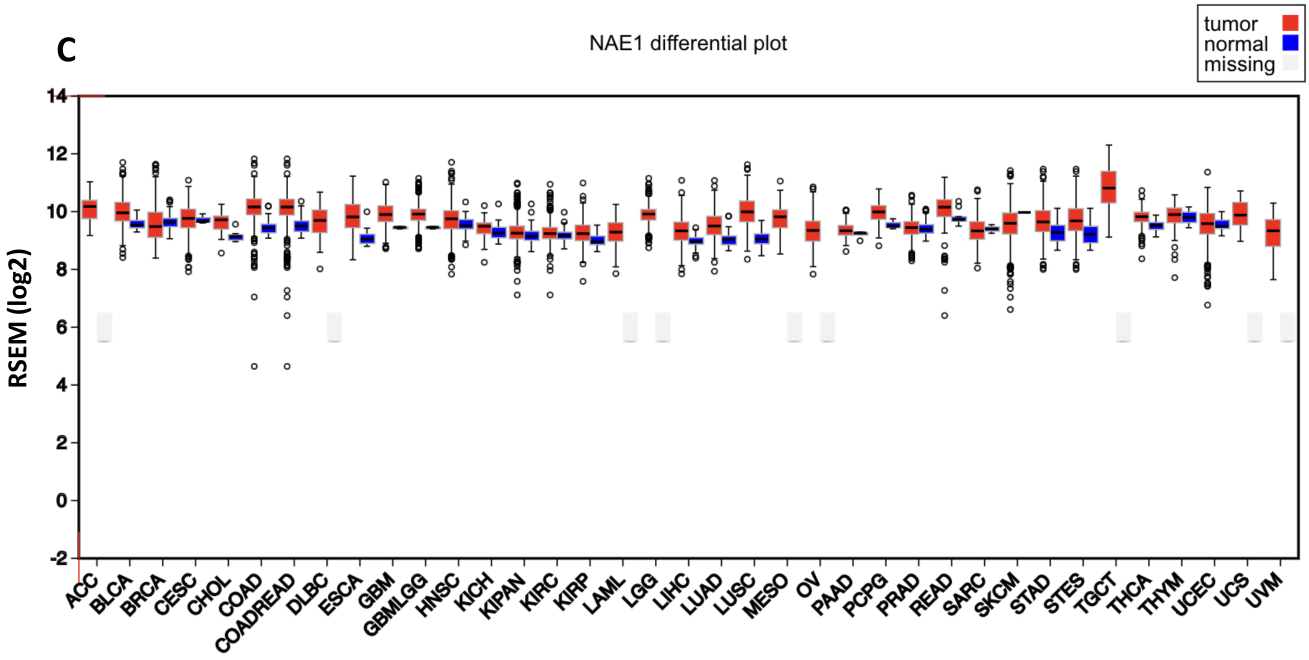

Supplementary Figure S5: NAE1 gene expression in TGCT and normal testis tissue. (A) Meta-analysis of Affymetrix microarray data from tissues (*n = 3*). Data represent independent biological replicate mean ± SD. (B) UCSC Xena browser-mediated analysis of NAE1 isoform expression in TGCT tissues (purple) and normal testis tissue (green). (C) Comparison of NAE1 expression in different tumor entities (red) and normal tissues (blue) using the Firebrowse database.

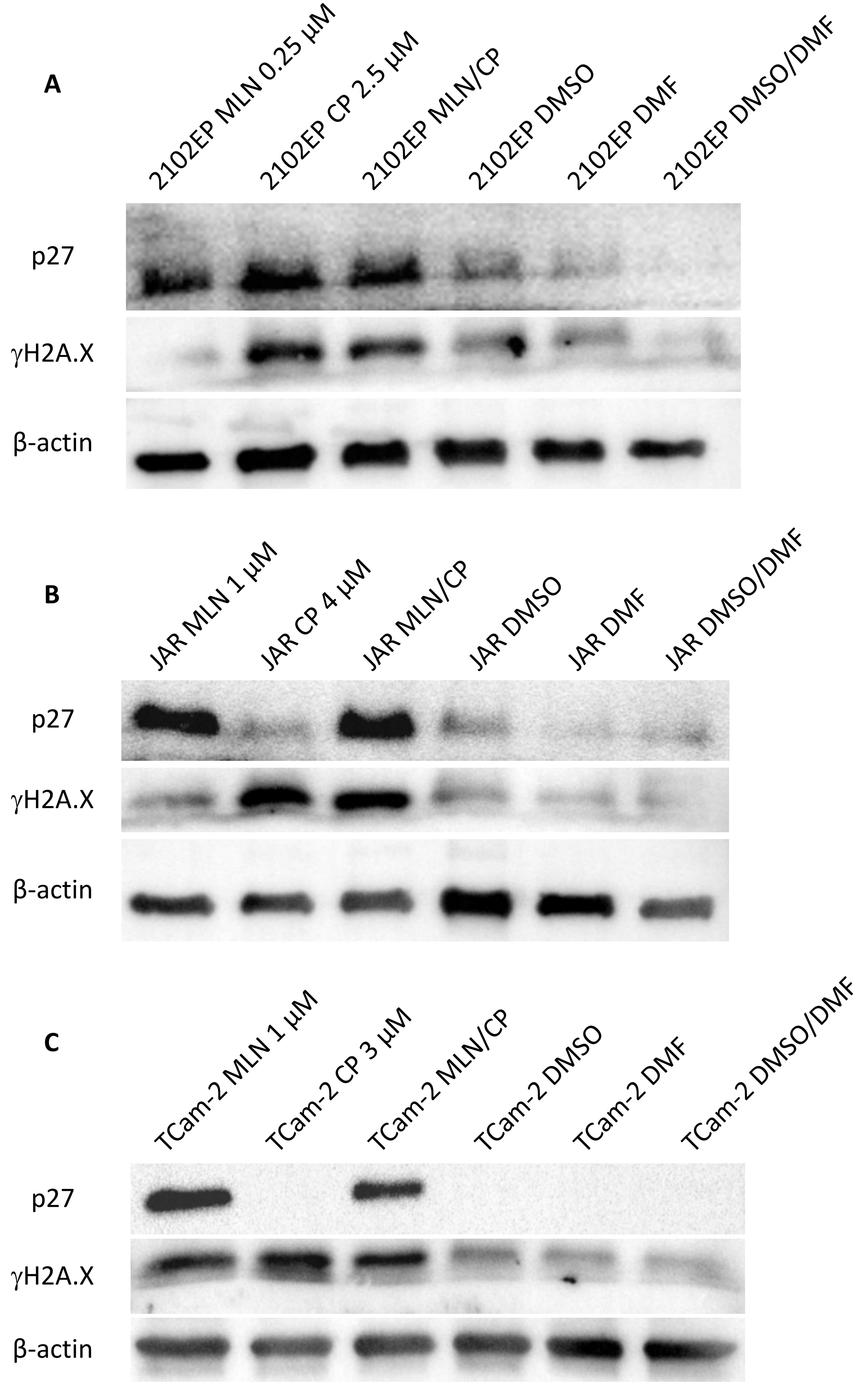

Supplementary Figure S6: Mode of action of MLN4924 (MLN) and cisplatin (CP). (A) 2102EP, (B) JAR and (C) TCam-2 cells were seeded in 6 well cell culture plates ($1.5x10^5$ cells/well) and treated for 2 days with MLN4924, cisplatin, combination of both drugs using indicated concentrations or corresponding solvent control (DMSO, DMF, DMSO/DMF). Subsequently, proteins were isolated and analyzed via SDS-PAGE and Western Blot using p27, γH2A.X and β-actin antibodies.

A

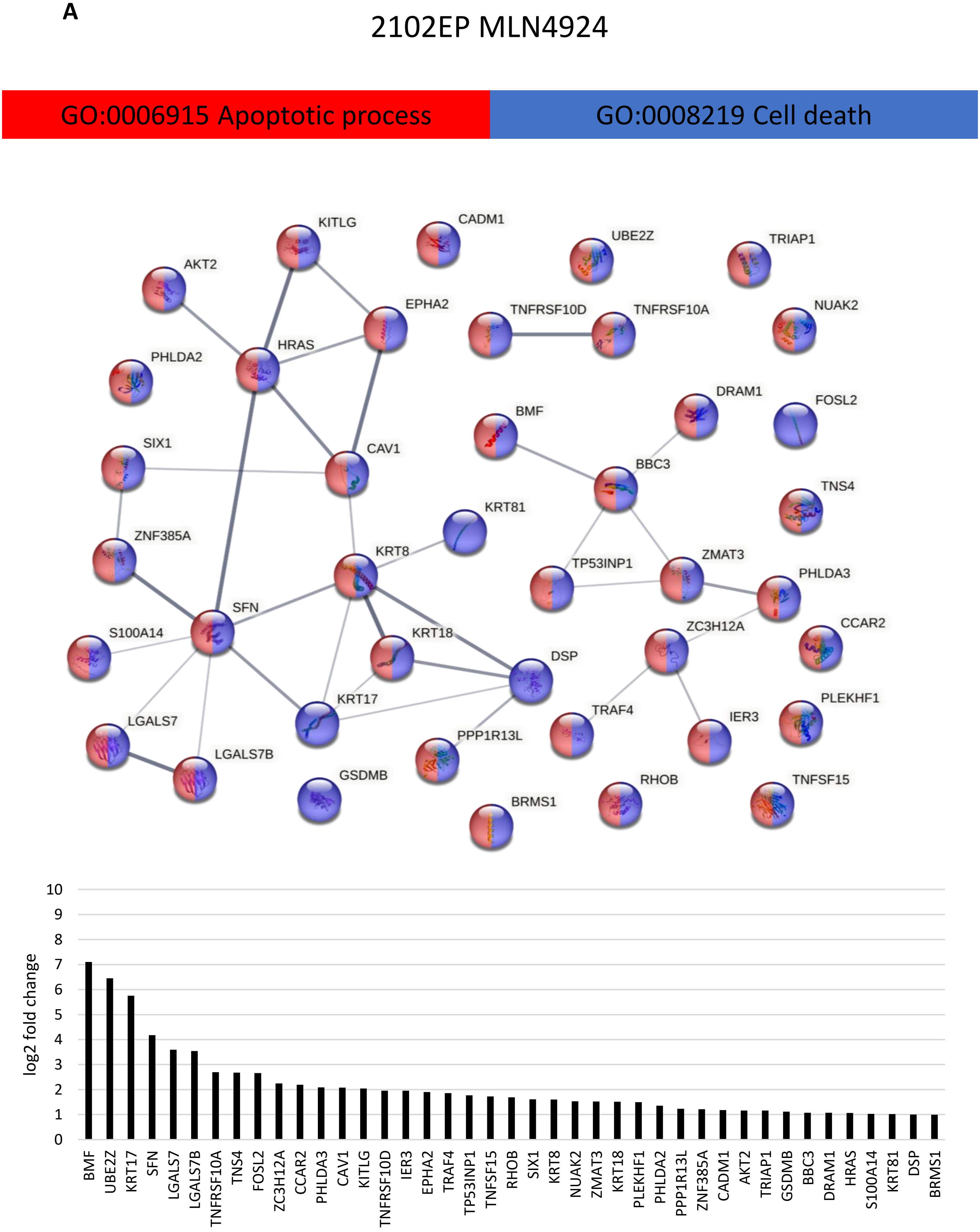

Supplementary Figure S7: Complete STRING network of connected and non-connected differentially upregulated genes associated with GO terms apoptosis and cell death. Additionally, log2 fold changes are depicted. Transcriptome analysis of 2102EP cells after 2 days of (A) MLN4924 (0.25 µM), (B) cisplatin (2.5 µM) or (C) MLN4924/cisplatin combination (0.25 µM/2.5 µM) treatment. Data represent independent biological replicate mean. For all samples *n* = 3.

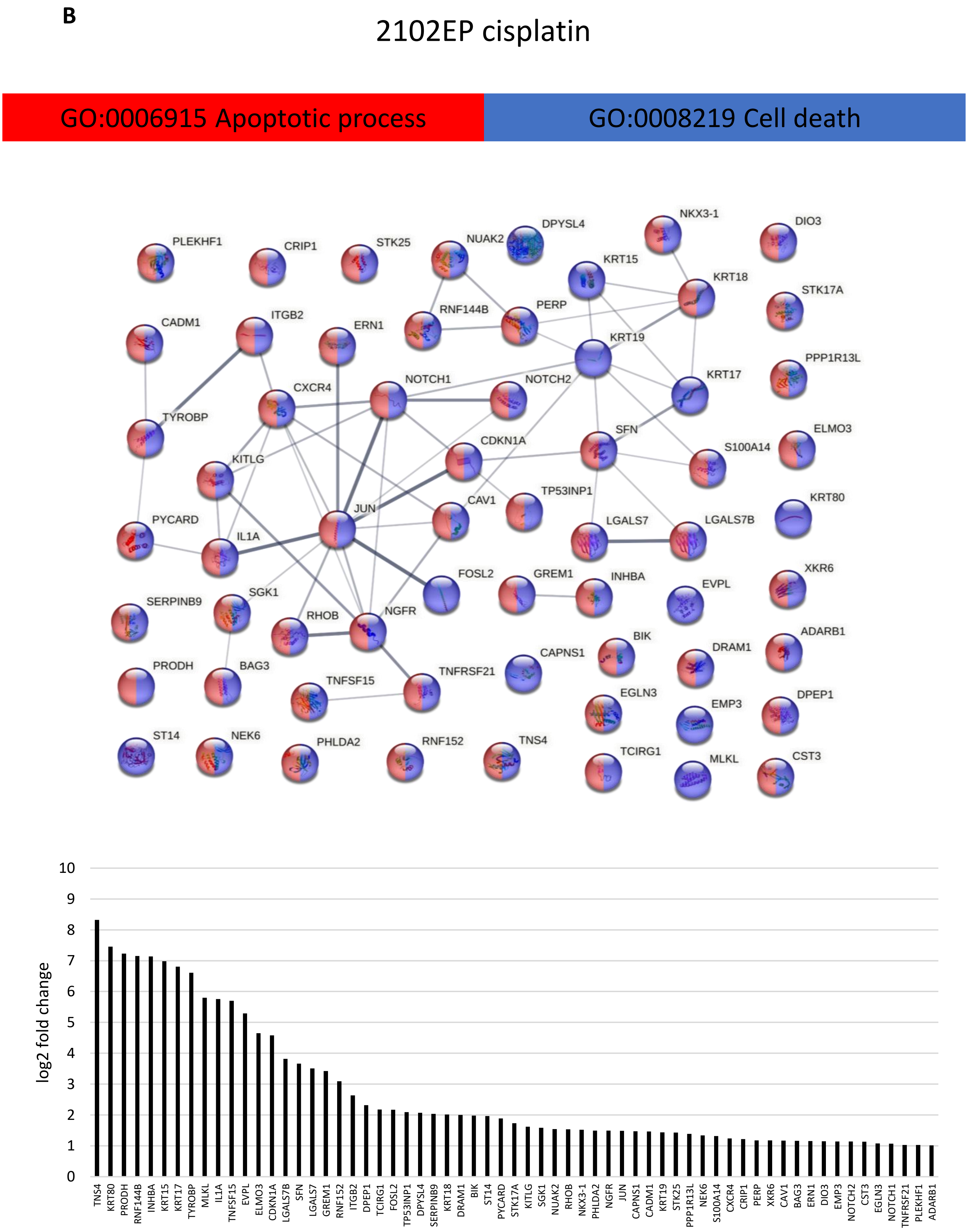

Supplementary Figure S7: Complete STRING network of connected and non-connected differentially upregulated genes associated with GO terms apoptosis and cell death. Additionally, log2 fold changes are depicted. Transcriptome analysis of 2102EP cells after 2 days of (A) MLN4924 (0.25 µM), (B) cisplatin (2.5 µM) or (C) MLN4924/cisplatin combination (0.25 µM/2.5 µM) treatment. Data represent independent biological replicate mean. For all samples *n* = 3.

C

2102EP MLN4924/cisplatin

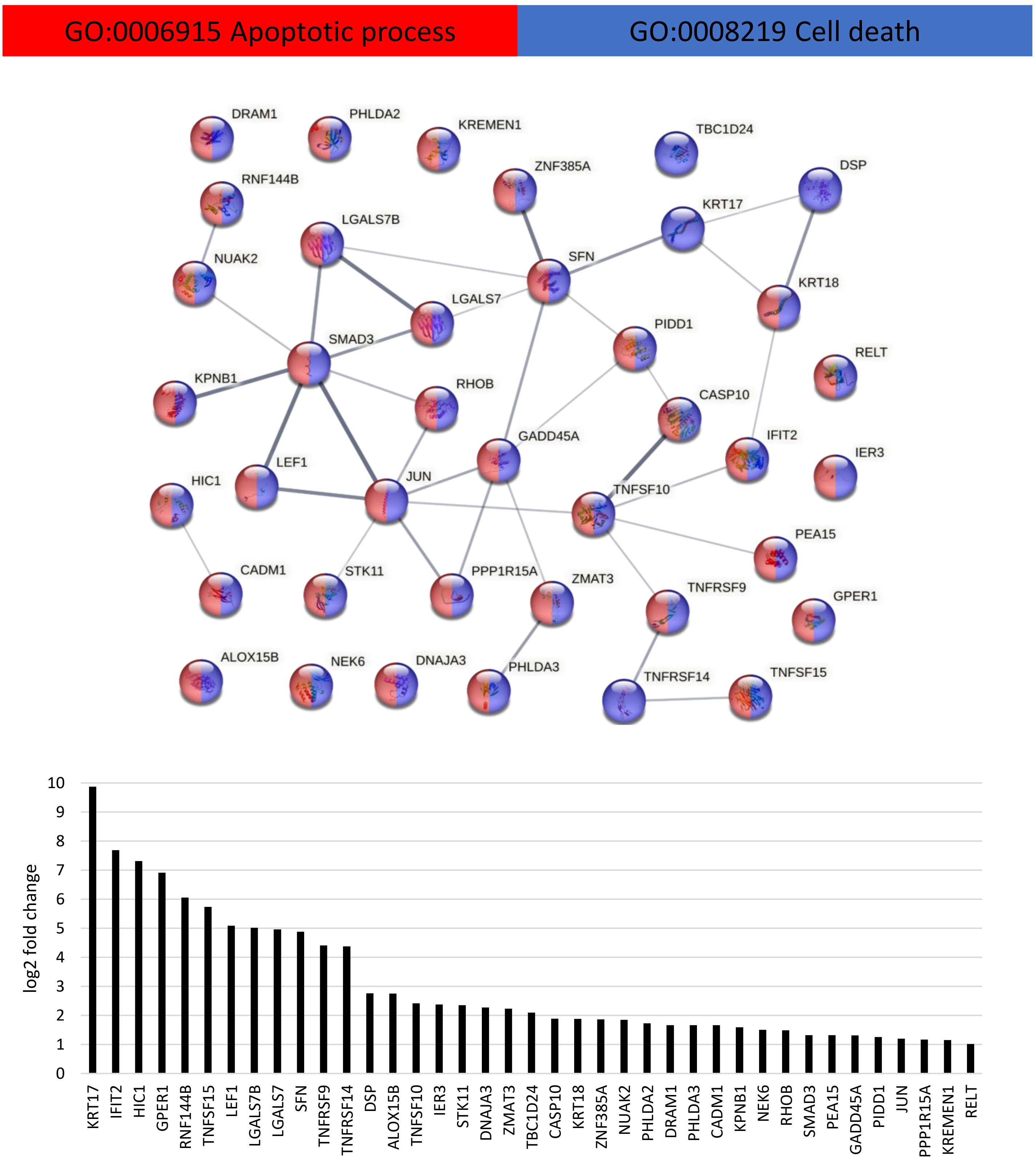

Supplementary Figure S7: Complete STRING network of connected and non-connected differentially upregulated genes associated with GO terms apoptosis and cell death. Additionally, log2 fold changes are depicted. Transcriptome analysis of 2102EP cells after 2 days of (A) MLN4924 (0.25 µM), (B) cisplatin (2.5 µM) or (C) MLN4924/cisplatin combination (0.25 µM/2.5 µM) treatment. Data represent independent biological replicate mean. For all samples *n* = 3.

D

JAR MLN4924

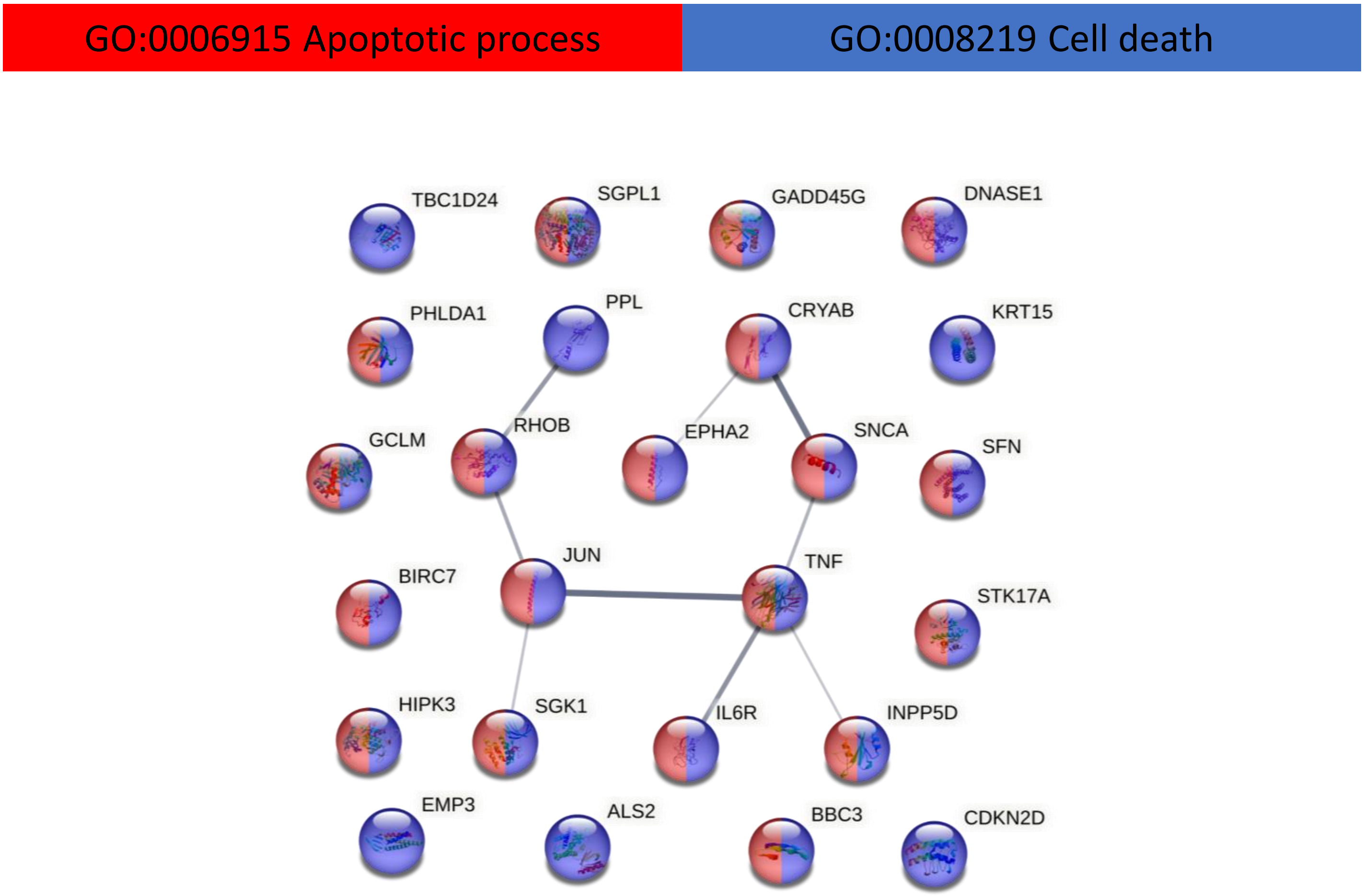

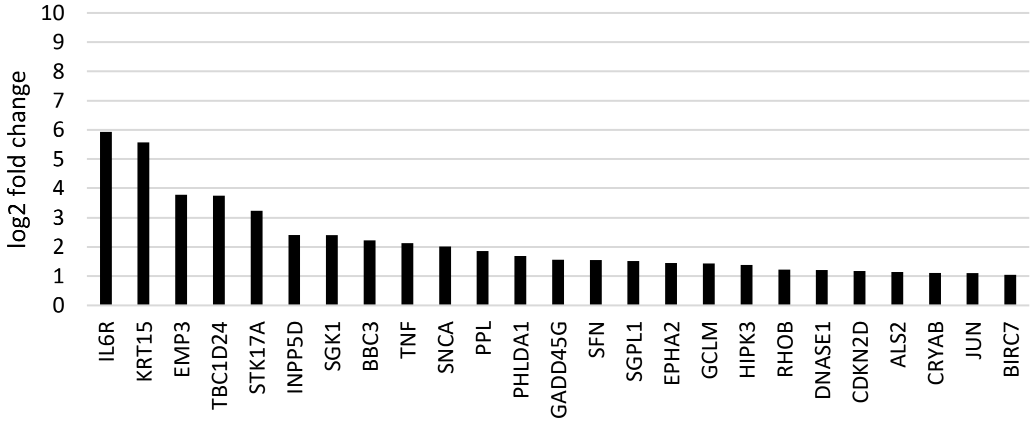

Supplementary Figure S7: Complete STRING network of connected and non-connected differentially upregulated genes associated with GO terms apoptosis and cell death. Additionally, log2 fold changes are depicted. Transcriptome analysis of JAR cells after 2 days of (D) MLN4924 (1 µM), (E) cisplatin (4 µM) or (F) MLN4924/cisplatin combination (1 µM/4 µM) treatment. Data represent independent biological replicate mean. For all samples *n* = 3.

E

JAR cisplatin

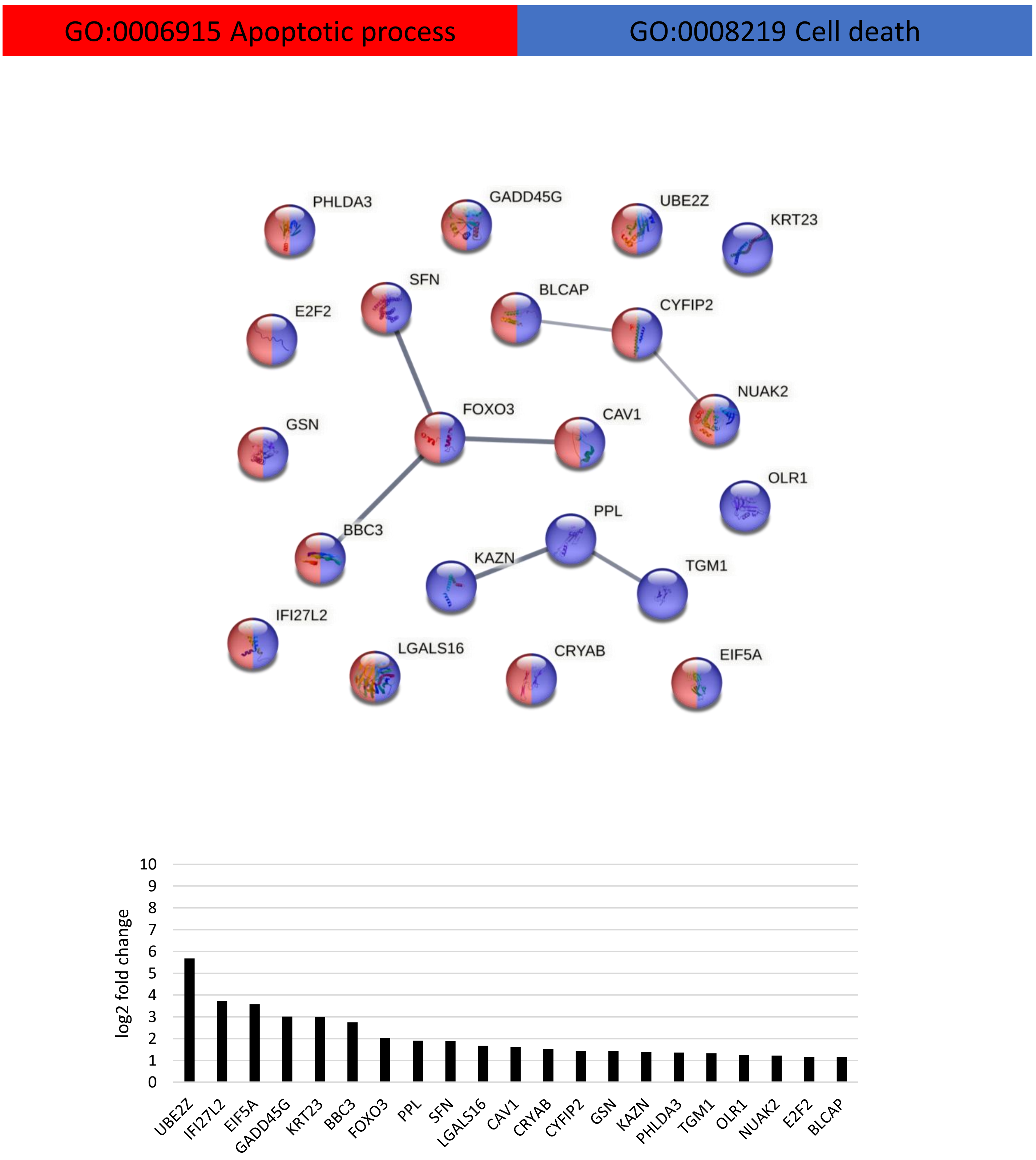

Supplementary Figure S7: Complete STRING network of connected and non-connected differentially upregulated genes associated with GO terms apoptosis and cell death. Additionally, log2 fold changes are depicted. Transcriptome analysis of JAR cells after 2 days of (D) MLN4924 (1 µM), (E) cisplatin (4 µM) or (F) MLN4924/cisplatin combination (1 µM/4 µM) treatment. Data represent independent biological replicate mean. For all samples *n* = 3.

F

JAR MLN4924/cisplatin

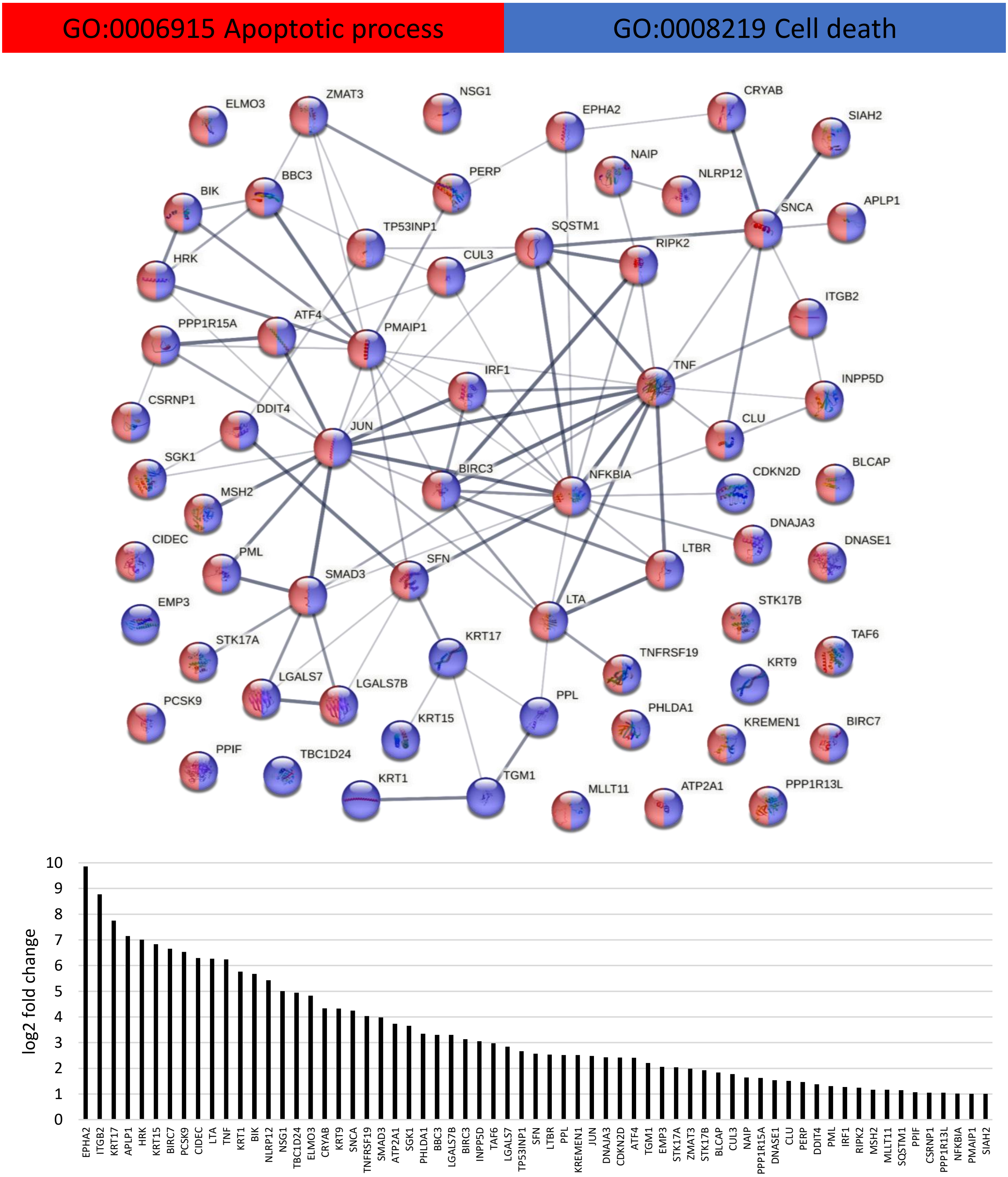

Supplementary Figure S7: Complete STRING network of connected and non-connected differentially upregulated genes associated with GO terms apoptosis and cell death. Additionally, log2 fold changes are depicted. Transcriptome analysis of JAR cells after 2 days of (D) MLN4924 (1 µM), (E) cisplatin (4 µM) or (F) MLN4924/cisplatin combination (1 µM/4 µM) treatment. Data represent independent biological replicate mean. For all samples *n* = 3.

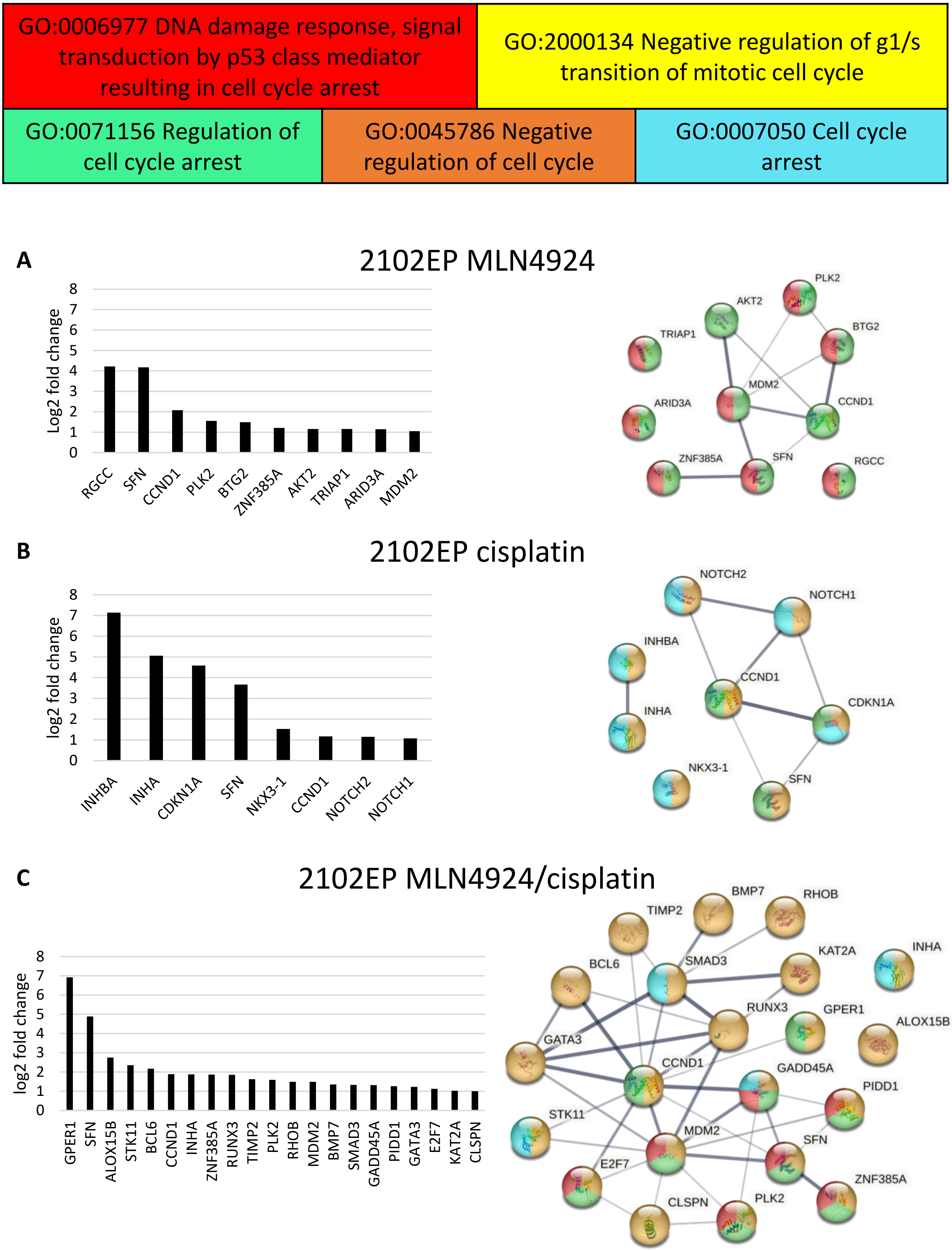

Supplementary Figure S8: Transcriptome analysis revealed cell cycle associated GO terms and STRING interaction networks. Log2 fold change of differentially deregulated genes related to the cell cycle is shown. Transcriptome analysis of 2102EP cells after 2 days of (A) MLN4924 (0.25 µM), (B) cisplatin (2.5 µM) or (C) MLN4924/cisplatin combination (0.25 µM/2.5 µM) treatment. Data represent independent biological replicate mean. For all samples n = 3.

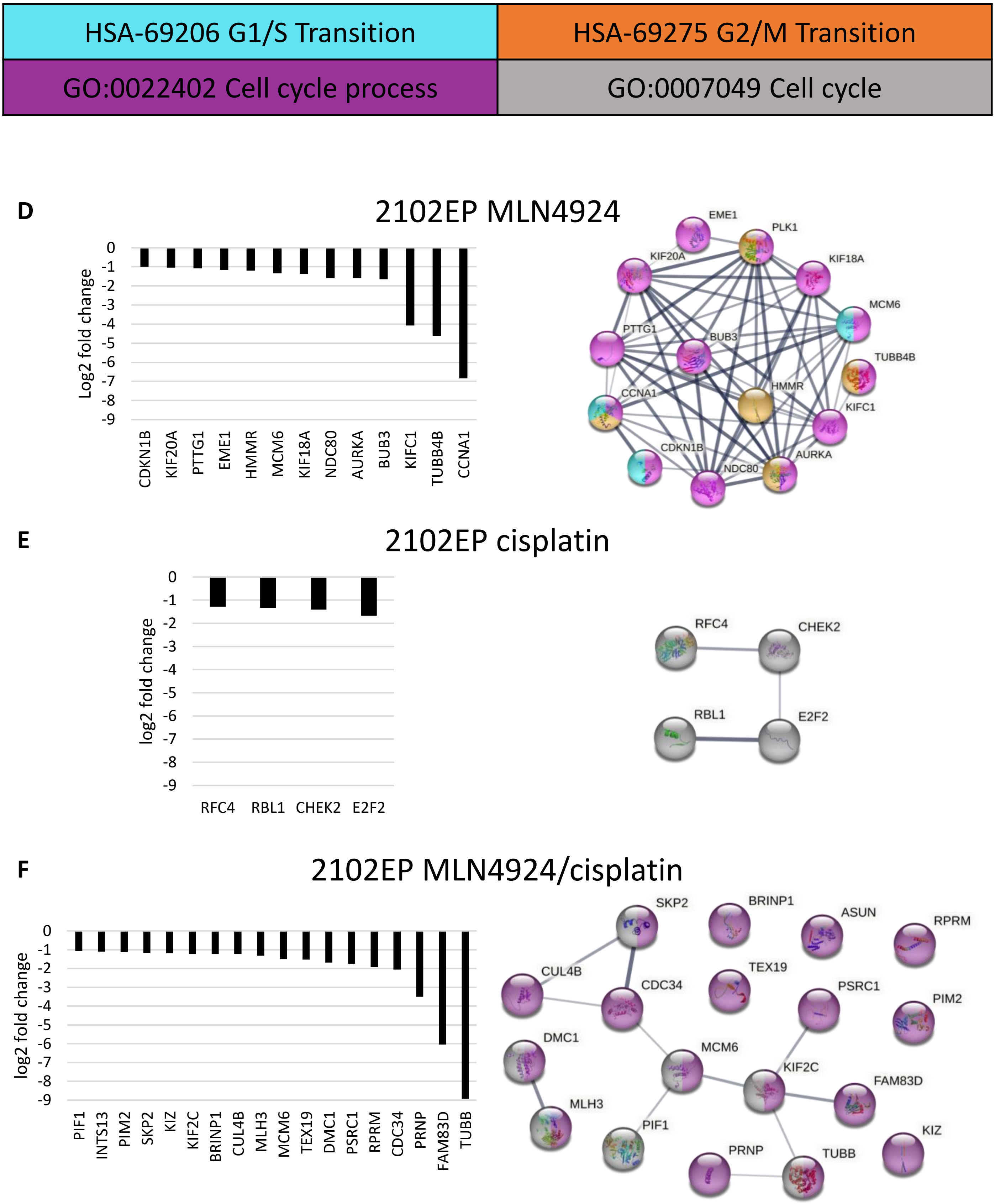

Supplementary Figure S8: Transcriptome analysis revealed cell cycle associated GO terms and STRING interaction networks. Log2 fold change of differentially deregulated genes related to the cell cycle is shown. Transcriptome analysis of 2102EP cells after 2 days of (D) MLN4924 (0.25 µM), (E) cisplatin (2.5 µM) or (F) MLN4924/cisplatin combination (0.25 µM/2.5 µM) treatment. Data represent independent biological replicate mean. For all samples *n* = 3.

GO:0006977 DNA damage response, signal transduction by p53 class mediator resulting in cell cycle arrest	GO:1901988 Negative regulation of cell cycle phase transition
GO:0071156 Regulation of cell cycle arrest	GO:2000134 Negative regulation of g1/s transition of mitotic cell cycle

A

JAR MLN4924

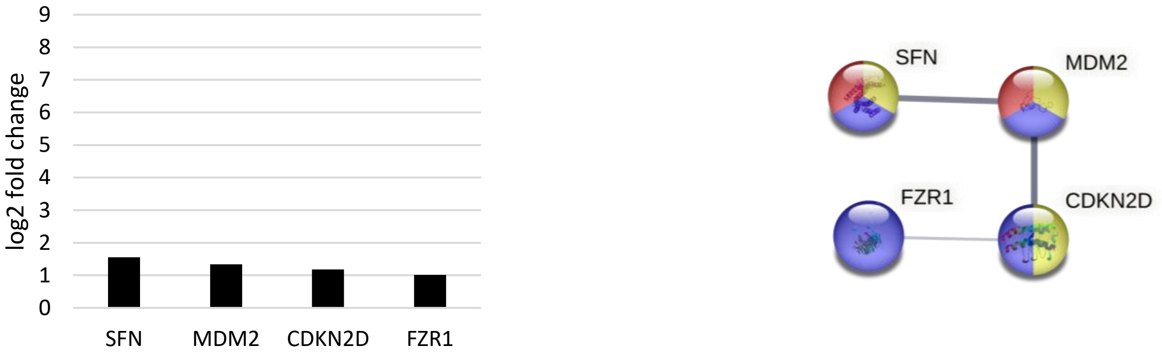

B

JAR cisplatin

No cell cycle related interaction network.

C

JAR MLN4924/cisplatin

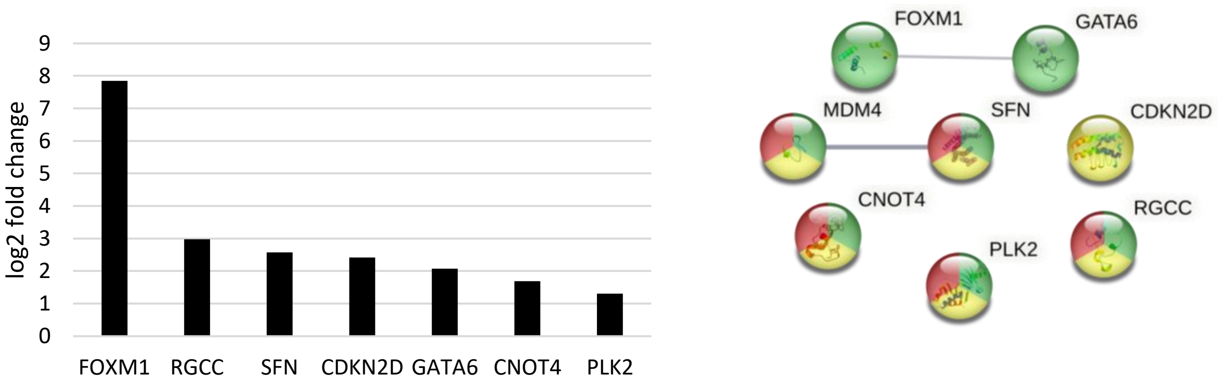

Supplementary Figure S9: Transcriptome analysis revealed cell cycle associated GO terms and STRING interaction networks. Log2 fold change of differentially deregulated genes related to the cell cycle is shown. Transcriptome analysis of JAR cells after 2 days of (A) MLN4924 (1 µM), (B) cisplatin (4 µM) or (C) MLN4924/cisplatin combination (1 µM/4 µM) treatment. Data represent independent biological replicate mean. For all samples *n* = 3.

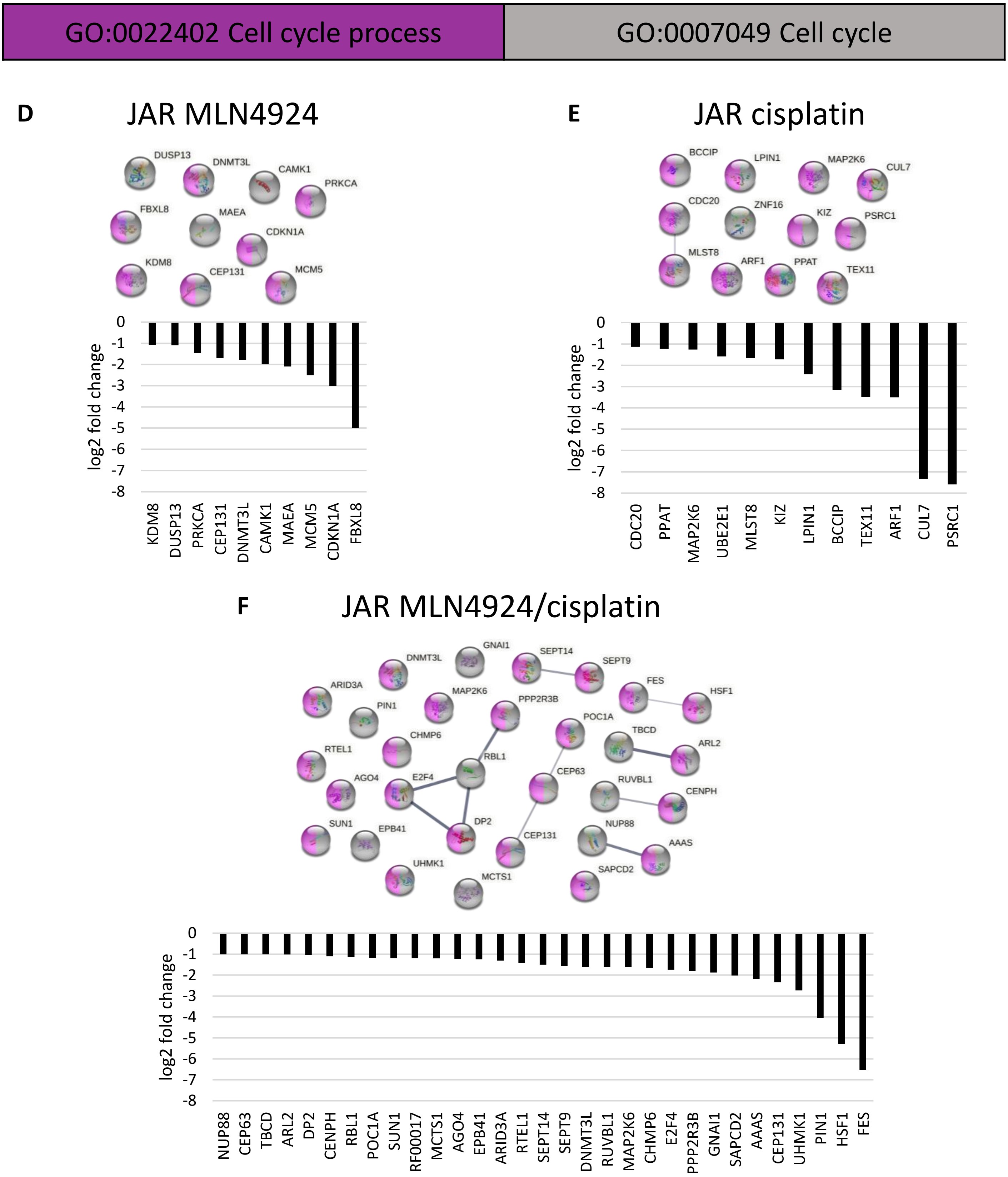

Supplementary Figure S9: Transcriptome analysis revealed cell cycle associated GO terms and STRING interaction networks. Log2 fold change of differentially deregulated genes related to the cell cycle is shown. Transcriptome analysis of JAR cells after 2 days of (D) MLN4924 (1 µM), (E) cisplatin (4 µM) or (F) MLN4924/cisplatin combination (1 µM/4 µM) treatment. Data represent independent biological replicate mean. For all samples *n* = 3.

A

2102EP MLN4924

GO:0030154 Cell differentiation

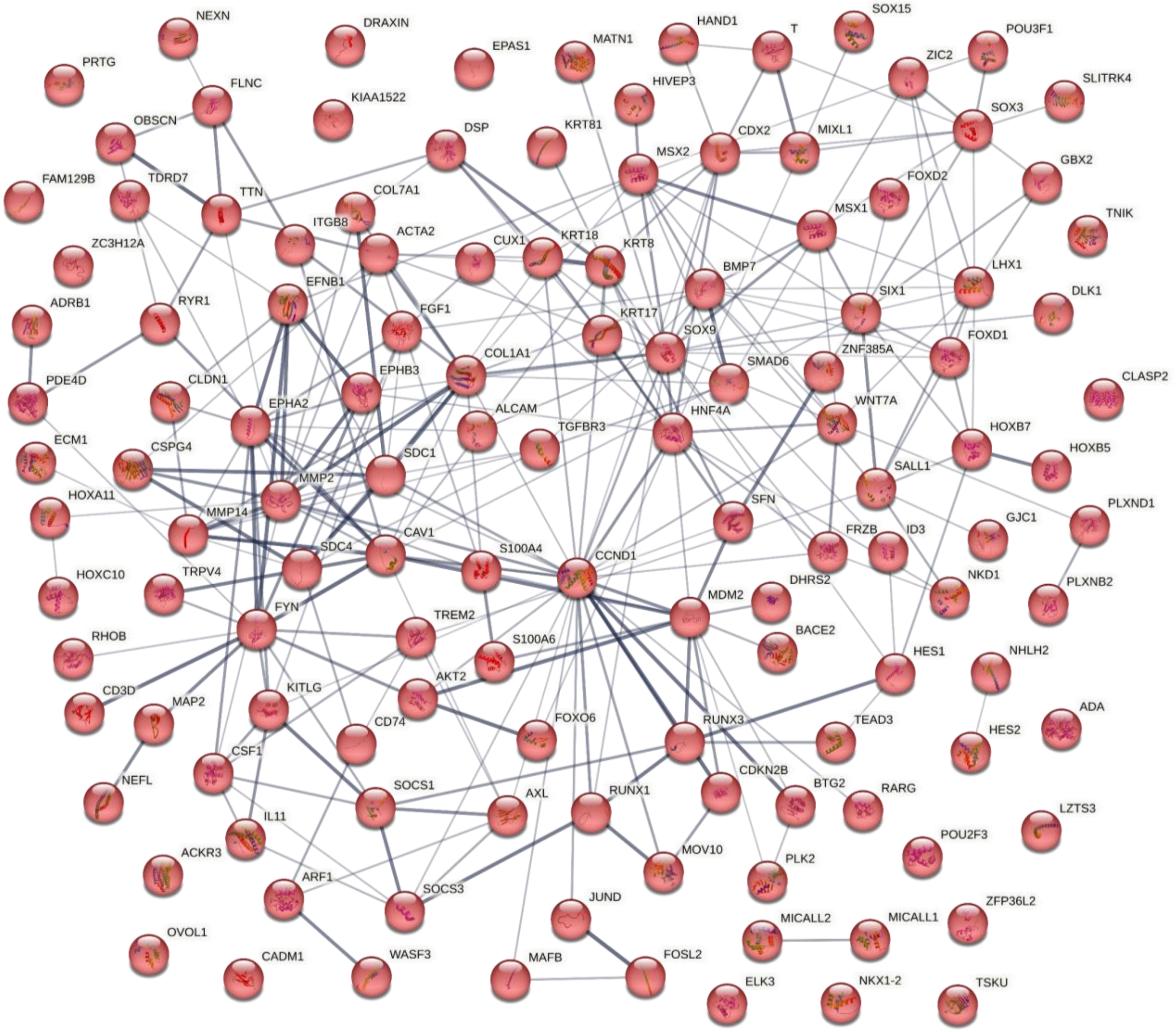

Supplementary Figure S10: Complete STRING network with all connected and unconnected protein interactions associated with GO term cell differentiation. Transcriptome analysis was performed on 2102EP cells after 2 days of (A) MLN4924 (0.25 µM), (B) cisplatin (2.5 µM) or (C) MLN4924/cisplatin combination (0.25 µM/2.5 µM) treatment. For all samples *n* = 3.

B

2102EP cisplatin

GO:0030154 Cell differentiation

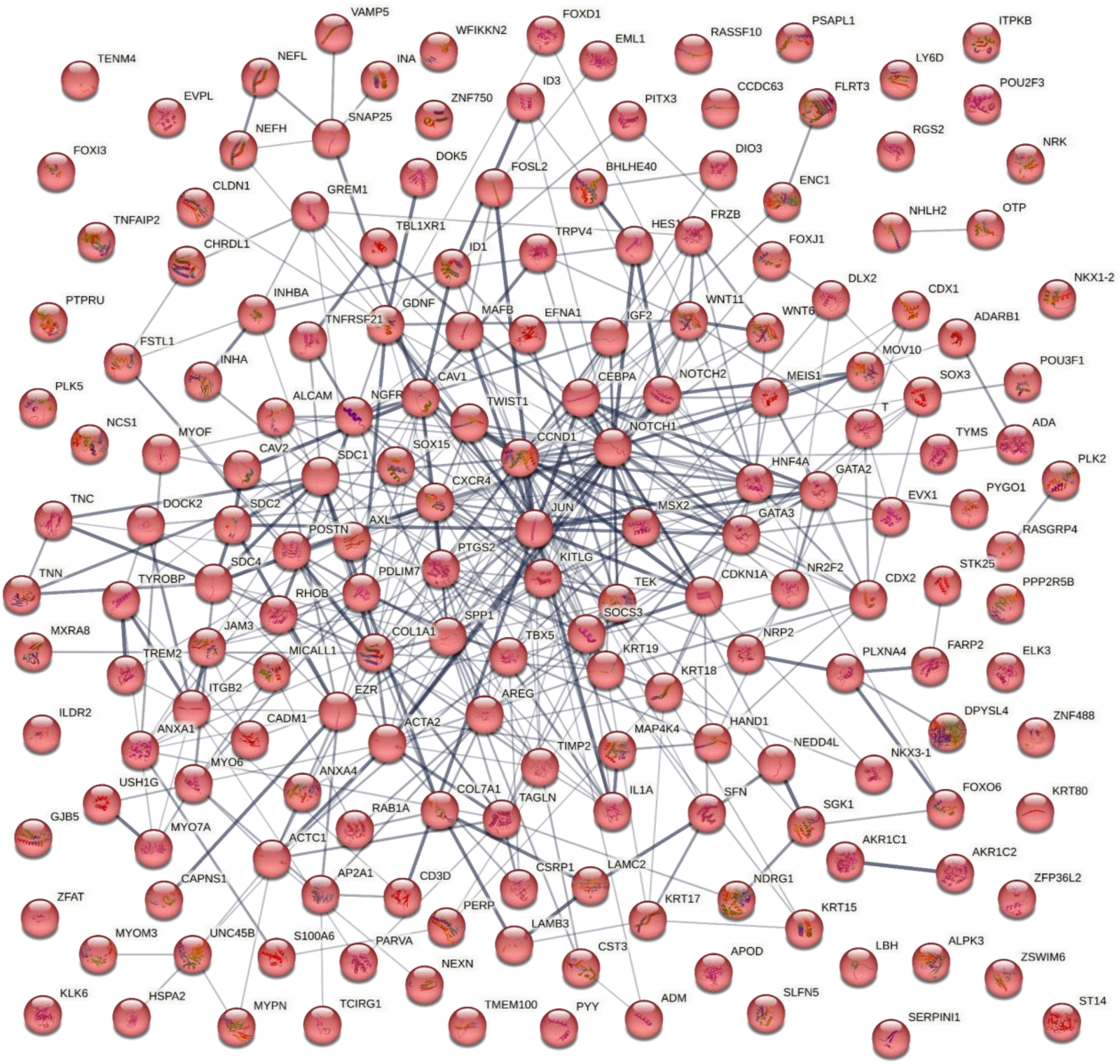

Supplementary Figure S10: Complete STRING network with all connected and unconnected protein interactions associated with GO term cell differentiation. Transcriptome analysis was performed on 2102EP cells after 2 days of (A) MLN4924 (0.25 µM), (B) cisplatin (2.5 µM) or (C) MLN4924/cisplatin combination (0.25 µM/2.5 µM) treatment. For all samples *n* = 3.

C

2102EP MLN4924/cisplatin

GO:0030154 Cell differentiation

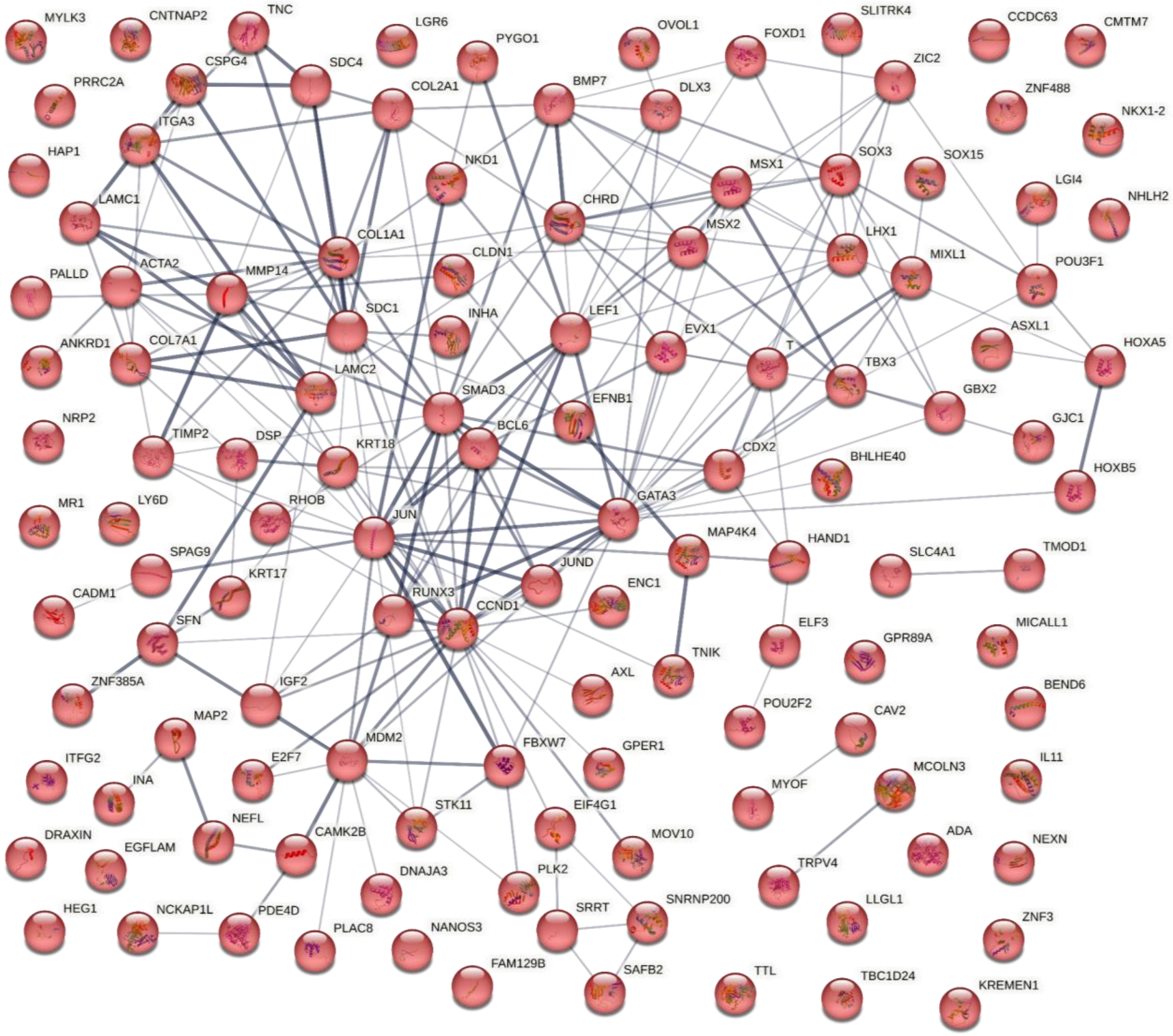

Supplementary Figure S10: Complete STRING network with all connected and unconnected protein interactions associated with GO term cell differentiation. Transcriptome analysis was performed on 2102EP cells after 2 days of (A) MLN4924 (0.25 µM), (B) cisplatin (2.5 µM) or (C) MLN4924/cisplatin combination (0.25 µM/2.5 µM) treatment. For all samples *n* = 3.

D

JAR MLN4924

GO:0030154 Cell differentiation

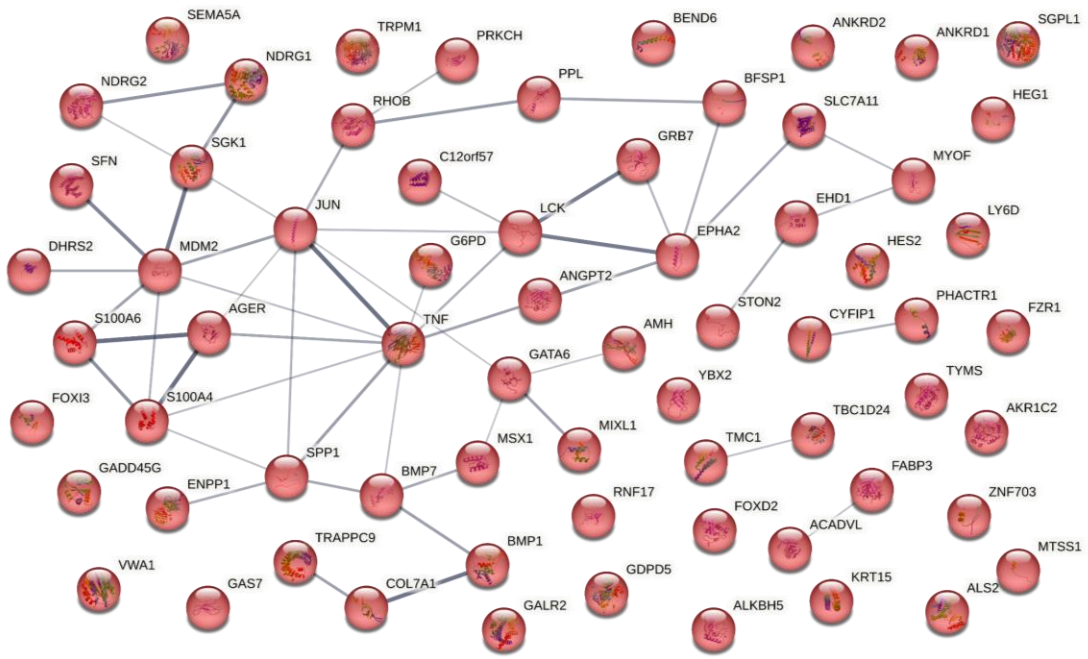

E

JAR cisplatin

GO:0030154 Cell differentiation

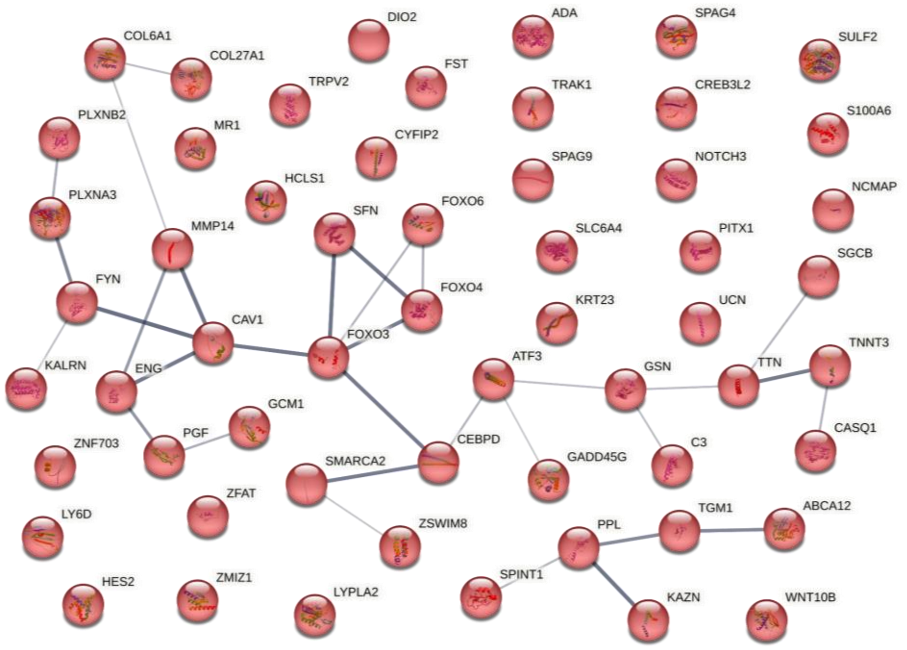

Supplementary Figure S10: Complete STRING network with all connected and unconnected protein interactions associated with GO term cell differentiation. Transcriptome analysis was performed on JAR cells after 2 days of (D) MLN4924 (1 µM), (E) cisplatin (4 µM) or (F) MLN4924/cisplatin combination (1 µM/4 µM) treatment. For all samples *n* = 3.

F

JAR MLN4924/cisplatin

GO:0030154 Cell differentiation

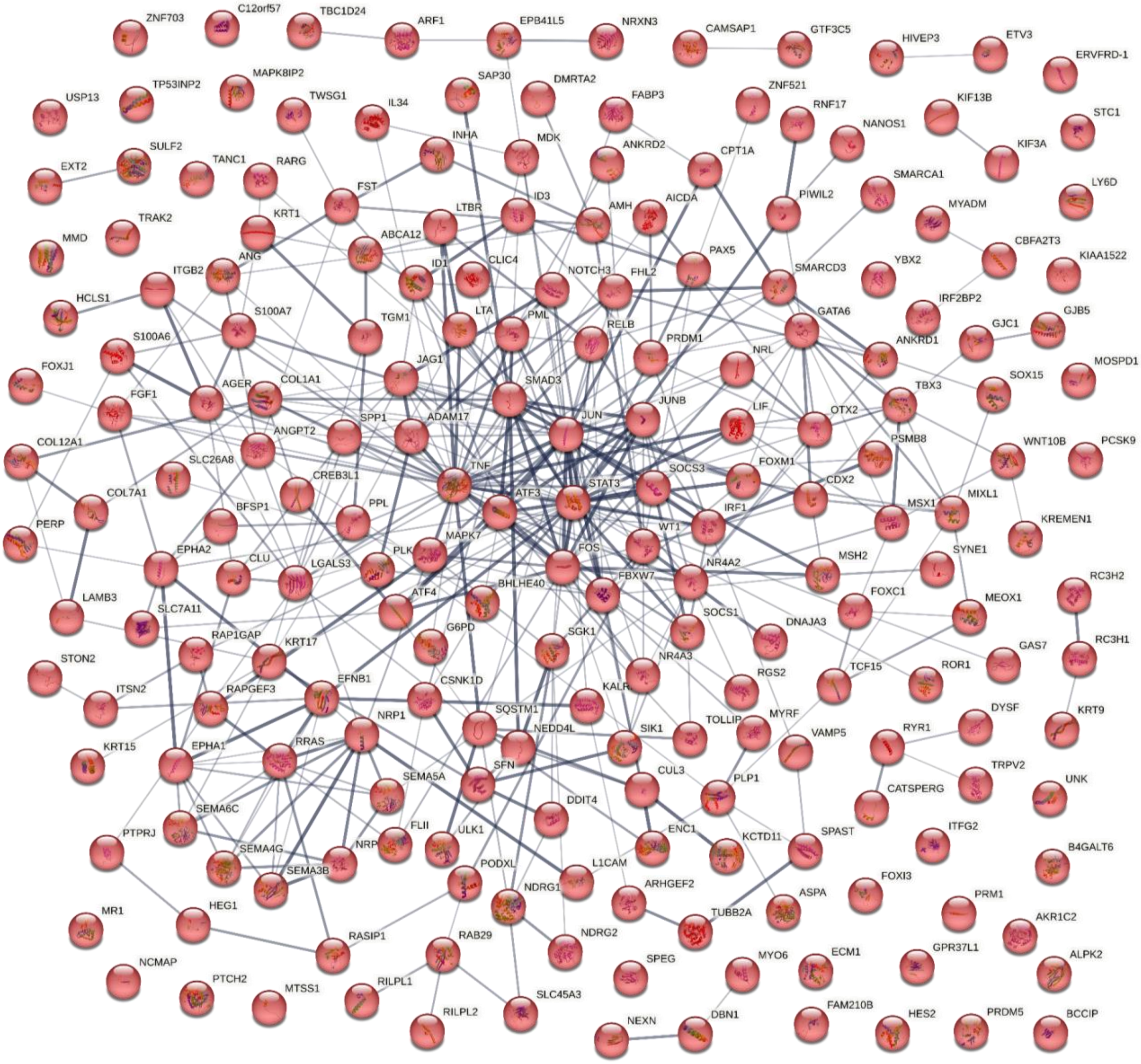

Supplementary Figure S10: Complete STRING network with all connected and unconnected protein interactions associated with GO term cell differentiation. Transcriptome analysis was performed on JAR cells after 2 days of (D) MLN4924 (1 µM), (E) cisplatin (4 µM) or (F) MLN4924/cisplatin combination (1 µM/4 µM) treatment. For all samples *n* = 3.

GO:0003700 DNA-binding transcription factor activity

A

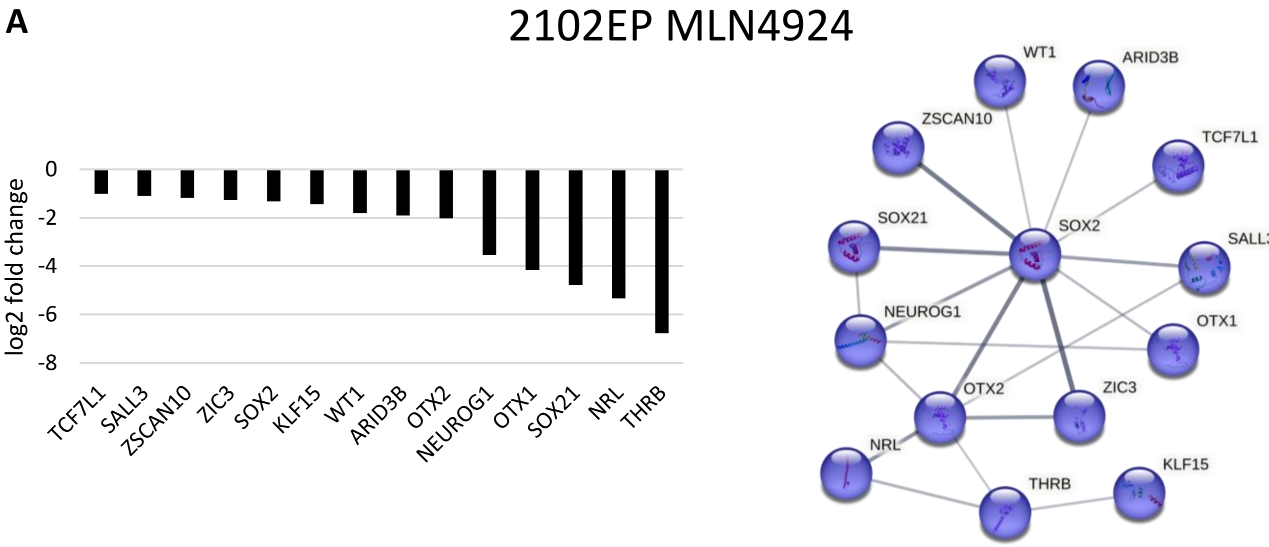

B

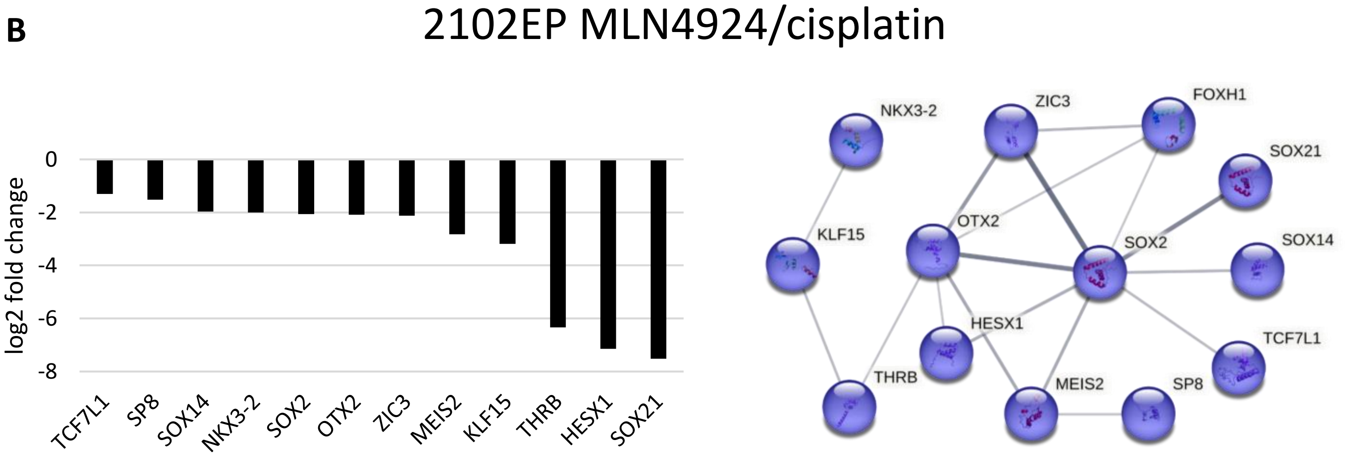

Supplementary Figure S11: Transcriptome analysis after MLN4924 only and MLN4924/cisplatin combination treatment revealed downregulation of transcription factors in 2102EP and JAR cells. Analysis was performed 2 days after treatment of (A) 2102EP MLN4924 (0.25 µM), (B) 2102EP MLN4924/cisplatin (0.25 µM/2.5 µM), (C) JAR MLN4924 (1 µM) and (D) JAR MLN4924/cisplatin (1 µM/4 µM). Data represent independent biological replicate mean. For all samples *n* = 3.

GO:0003700 DNA-binding transcription factor activity

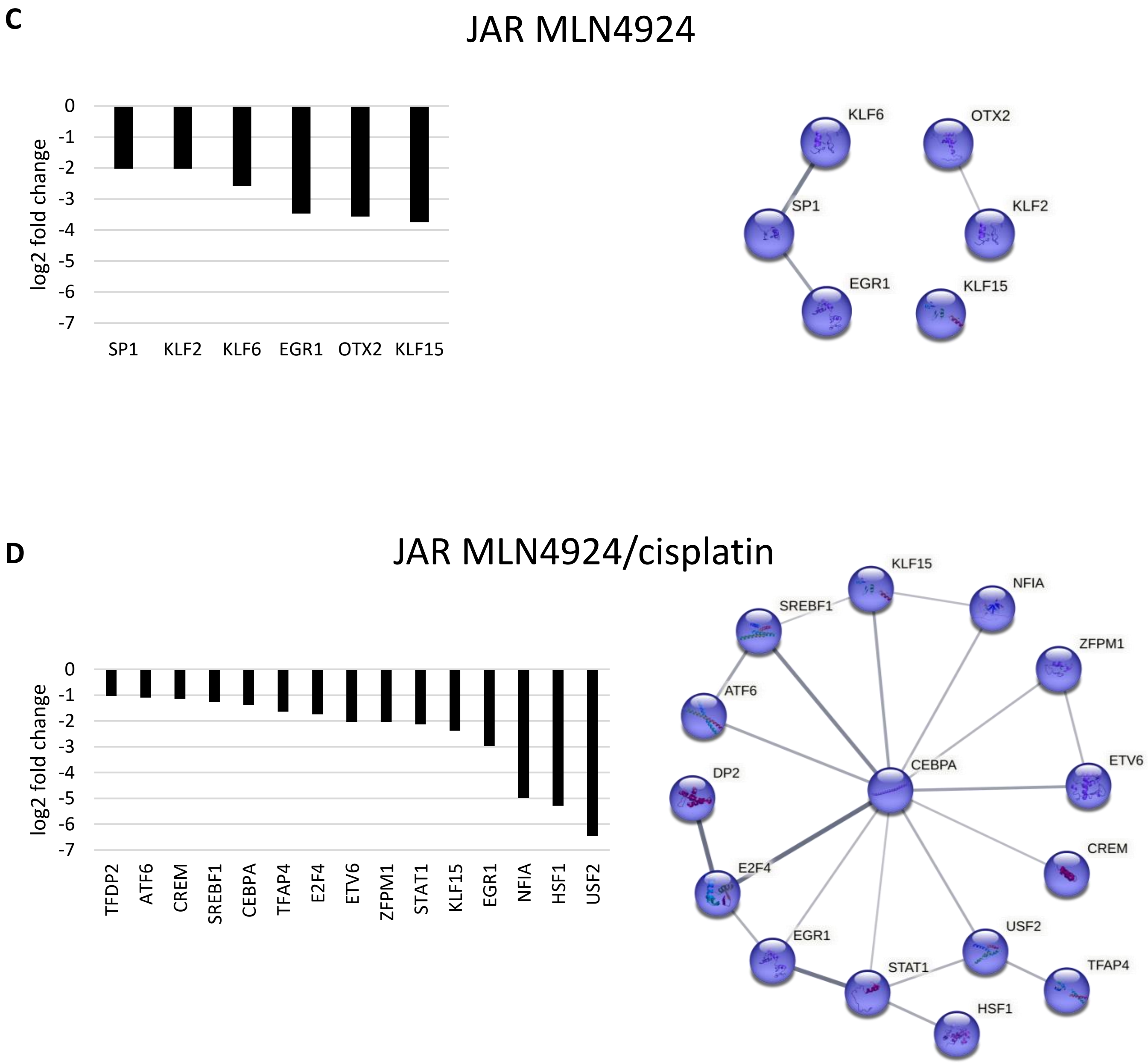

Supplementary Figure S11: Transcriptome analysis after MLN4924 only and MLN4924/cisplatin combination treatment revealed downregulation of transcription factors in 2102EP and JAR cells. Analysis was performed 2 days after treatment of (A) 2102EP MLN4924 (0.25 µM), (B) 2102EP MLN4924/cisplatin (0.25 µM/2.5 µM), (C) JAR MLN4924 (1 µM) and (D) JAR MLN4924/cisplatin (1 µM/4 µM). Data represent independent biological replicate mean. For all samples *n* = 3.
