# Supplementary material for: Genome-scale CRISPR screen reveals neddylation to contribute to cisplatin resistance of testicular germ cell tumours
Source: Br J Cancer. 2023 Apr 6;128(12):2270–82. doi: 10.1038/s41416-023-02247-5 (PMC10241889; doi:10.1038/s41416-023-02247-5)
Supplement: Supplementary file 2 — Supplementary Methods [file 41416_2023_2247_MOESM2_ESM.docx]

**Supplementary Methods**

*Cell culture*

2102EP, NCCIT and NT2/D1 cells were kindly provided by Prof. Dr. L. Looijenga (Princess Máxima Center for Pediatric Oncology, Utrecht, The Netherlands). Cisplatin resistant lines 2102EP-R, NCCIT-R, NT2/D1-R were a kind gift from PD Dr. F. Honecker (ZeTup Silberturm, St. Gallen, Switzerland). JAR cells were obtained from ATCC (Manassas, VA, USA). MPAFs were kindly provided by PD Dr. M. Peitz (IRN, Bonn University, Bonn, Germany).

*Lentivirus production (SAMv2 library) and concentration*

For lentivirus production, 2.1x10^7^ HEK293T cells were seeded in a T225 cell culture flask and transfected the next day with the helper plasmids pMD2.G and psPAX2 for virus assembly and either the SAMv2 plasmid library or the lentiMPHv2 plasmid using Transporter 5 Transfection Reagent (Polysciences, Warrington, PA, USA). 2 days after transfection cell culture supernatant was collected, filtered and concentrated using the Lenti-X concentrator according to the manufacturer’s protocol (Takara BIO INC., Kusatsu, Shiga, Japan).

*Determination of multiplicity of infection (MOI)*

2102EP and JAR cells (10^5^ cells/well) were seeded in 6-well cell culture plates for virus titer determination. Next day the cell culture supernatant was supplemented with 10 µg/ml polybrene (Merck, Darmstadt, Germany) and 0, 15.63, 31.25, 62.5, 125 or 250 µl virus were added. Subsequently, cells were centrifuged at 1500 xg, 32 °C for 30 min to facilitate transduction. After 48 h of incubation and two PBS washing steps in between 1500 cells of each condition were seeded per well as quadruplicate in black walled-96-well plates. Two wells of each condition were treated with antibiotics (5 µg/ml blasticidin (Thermo Fisher Scientific, Waltham, USA) or 250 µg/ml hygromycin (Santa Cruz, Dallas, USA)). After all cells of the control had died cell viability was determined using CellTiter-Glo™ Luminescent Cell Viability Assay Kit (Promega, Madison, WI, USA) according to the manufacturer. MOI was calculated for each condition by referring the viability of treated sample to the untreated sample.

**Supplementary Tables**

Supplementary Table S1: Plasmids for genome-scale CRISPR/Cas9 activation screen

| **Plasmid** | **Plasmid components** | **Depositor** |
| --- | --- | --- |
| Human CRISPR Activation Library (SAMv2) | 70290 different sgRNAs for gene activation, dCas9 VP64 fusion protein, blasticidin resistance | Human CRISPR activation pooled library (SAMv2) was a gift from Feng Zhang (Addgene #1000000078) |
| lentiMPH v2 | MS2-P65-HSF1 activator helper complex, hygromycin resistance | lentiMPH v2 was a gift from Feng Zhang (Addgene plasmid # 89308) |
| pMD2.G | VSV-G envelope expressing plasmid | pMD2.G was a gift from Didier Trono (Addgene plasmid # 12259) |
| psPAX2 | Lentiviral packaging plasmid | psPAX2 was a gift from Didier Trono (Addgene plasmid # 12260) |

Supplementary Table S2: Cell numbers for genome-scale CRISPR/Cas9 activation screen

| Cell line | Library | Library size (sgRNAs) | Cell number for coverage of 100% ^1)^ | Cells for trans-duction | Coverage in the screen [%] | Cell number for gDNA isolation ^2)^ |
| --- | --- | --- | --- | --- | --- | --- |
| 2102EP^MPHv2^ | SAMv2 | 70290 | 1.17x10^8^ | 1.18 x10^8^ | 100.9 | 3.54x10^7^ |
| JAR^MPHv2^ | SAMv2 | 70290 | 1.17x10^8^ | 1.2x10^8^ | 102.6 | 3.61x10^7^ |

1. Coverage of 100% at MOI of 0.3 and 500 cells/sgRNA
2. To maintain coverage of 100% gDNA had to be isolated from 500 cells/sgRNA

Supplementary Table S3: Primary and secondary antibodies used in this study.

| **Target** | **Company** | **Species** | **Dilution** | **Order No.** |
| --- | --- | --- | --- | --- |
| NAE1 | Cell signaling, USA | Rabbit | 1:1000 | 14321S |
| GFP | Santa Cruz, Dallas, USA | Mouse | 1:1000 | sc9996 |
| P27 Kip1 | Cell signaling, USA | Rabbit | 1:1000 | 3686S |
| H2A.X pS139 (γH2A.X) | Abcam, Cambridge, UK | Rabbit | 1:2000 | ab11174 |
| β-Actin | Sigma-Aldrich (Merck), Germany | Mouse | 1:10000 | A5441 |
| Anti-mouse HRP | Agilent Technologies (Dako), USA | Rabbit | 1:750 | P0260 |
| Anti-rabbit HRP | Agilent Technologies (Dako), USA | Goat | 1:2000 | P0447 |
